# Adipocytes as a Mechanistic Link Between Incretin-Based Therapies and Hair Loss

**DOI:** 10.3390/biology15181642

**Published:** 2026-09-17

**Authors:** Aditya K. Gupta, Jaspreet Kaur, Vincent Piguet, Paradi Mirmirani

**Affiliations:** 1 Division of Dermatology, Department of Medicine, University of Toronto, Toronto, ON M5S 3H2, Canada; 2 Department of Medicine Dermatology, Women’s College Hospital, Toronto, ON M5S 1B2, Canada; 3 Independent Researcher, London, ON N5X 2P1, Canada; 4 Department of Dermatology, The Permanente Medical Group, Vallejo, CA 94589, USA; 5 Department of Dermatology, University of California, San Francisco, CA 94115, USA

**Keywords:** androgenetic alopecia, dermal adipocytes, glucagon-like peptide-1 receptor agonists, hair follicle, hair loss, incretins, telogen effluvium

## Abstract

The physiological effects of glucagon-like peptide-1 receptor agonists (GLP-1RAs) and dual glucose-dependent insulinotropic polypeptide (GIP)/GLP-1RAs extend beyond glucose metabolism and body weight regulation. Accumulating clinical evidence has highlighted an association between these incretin-based therapies and hair loss; however, the underlying molecular mechanisms are not fully understood. White adipose tissue (WAT) is increasingly recognized as a critical regulator of hair follicle homeostasis that may undergo substantial drug-associated functional alterations. Thus, GLP-1RAs and GIP/GLP-1RAs may indirectly modulate hair follicle biology and promote hair loss through changes in adipocyte function. This review assesses the effects of GLP-1RAs and GIP/GLP-1RAs on WAT, synthesizing findings from cellular systems, animal models, and human clinical research. Collectively, these therapies may influence adipocyte differentiation, adipokine secretion, and pro-inflammatory cytokine signaling in a context-dependent manner; however, most data are derived from subcutaneous WAT and have yet to be established in the distinct dermal WAT compartment that surrounds hair follicles. Accordingly, we propose a hypothetical, non-exclusive model in which drug-associated adipocyte reprogramming may alter the hair follicle microenvironment and disrupt normal follicular cycling, potentially contributing to increased hair shedding. Validation of this potential pathway is required and may inform the development of targeted strategies to prevent or treat this adverse event.

## Simple Summary

Incretin-based medications used to treat type 2 diabetes and obesity have been linked to hair loss, but the reasons for this side effect remain unclear. In the skin, fat cells (adipocytes) located around hair follicles provide signals that are important for normal hair growth and they may be affected by these medications. This narrative review examines the existing literature regarding the direct effects of incretin-based medications on adipocytes and evaluates whether changes in cellular function could provide one biological explanation for treatment-associated hair loss. Current evidence suggests that in specific contexts, incretin-based medications may alter adipocyte development and function, including the release of adipokines and cytokines (signaling proteins that regulate communication between cells). These changes may contribute to drug-associated hair loss. However, available evidence comes from experimental models that do not specifically examine specialized adipocytes surrounding human hair follicles. Thus, the adipocyte-mediated pathways identified here as potential explanations for the association between incretin-based medications and hair loss have not been directly established and warrant further study. By identifying adipocytes as a possible link, this work highlights important priorities for future research, and may help inform the development of strategies to prevent or treat hair loss in patients receiving incretin-based medications.

## 1. Introduction

Glucagon-like peptide-1 receptor agonists (GLP-1RAs), including exenatide (synthetic exendin-4), semaglutide, and liraglutide, have demonstrated substantial clinical benefits, particularly in glycemic control and body weight reduction [1,2]. More recently, dual glucose-dependent insulinotropic polypeptide (GIP)/GLP-1RAs, such as tirzepatide, have demonstrated superior metabolic efficacy to GLP-1RAs alone [1,2]. Consequently, prescription of these incretin-based drugs has begun to expand to a broader range of patient populations [3,4]. Thus, defining the full spectrum of the drugs’ biological effects remains an important clinical priority.

The widespread adoption of incretin-based treatments has revealed hair loss as an increasingly reported concern in patients receiving GLP-1RAs or GIP/GLP-1RAs [5,6,7], prompting questions regarding whether these incretin-based drugs directly influence hair follicles. Clinically, hair loss typically manifests as androgenetic alopecia (AGA) or telogen effluvium (TE) [5,6,8]. While hair loss has been broadly attributed to rapid weight loss and nutritional factors in these patients [5,9], the molecular pathways contributing to this adverse event remain incompletely understood. Although recent reviews have characterized the clinical occurrence of hair loss or alopecia in patients [5,7,10], potential mechanisms have received comparatively limited attention. The present review moves beyond the clinical literature and broad explanations, taking a hypothesis-driven approach to examine preclinical evidence for plausible links between incretin-based therapies and hair loss.

Given the decrease in adipose tissue following GLP-1RA or GIP/GLP-1RA treatment [1], disruption of adipocyte homeostasis may represent one potential component of the multifactorial mechanisms underlying hair loss. Although historically viewed as a passive energy storage depot, white adipose tissue (WAT) is now recognized as a metabolically active structure involved in endocrine and immune functions, in part through the secretion of adipokines (such as leptin, adiponectin, resistin, and visfatin) and pro-inflammatory cytokines (including interleukin-6 [IL-6] and tumor necrosis factor alpha [TNFα]) [11,12]. Moreover, dermal WAT (dWAT) has been identified as a critical component of the hair follicle niche, where it influences stem cell activation and hair cycling [12,13,14,15,16,17,18].

Despite the key role of dermal adipocytes in hair follicle homeostasis, their potential association with drug-associated hair loss has not been sufficiently studied. Although no studies have yet directly demonstrated that GLP-1RAs or dual GIP/GLP-1RAs modify human dWAT, several lines of preclinical evidence suggest that these drugs may act directly on adipocytes. Adipocytes can express both GLP-1 receptors [19,20,21,22,23,24] and GIP receptors [24,25,26,27,28,29,30,31], through which incretin signaling may alter cellular differentiation and function. As these processes are involved in hair follicle cycling, adipocyte reprogramming by incretin-based therapies may represent one of several potential mechanisms contributing to hair loss. Therefore, this narrative review examines the current evidence supporting adipocyte-mediated pathways linking incretin-based therapies to hair loss, and highlights key gaps that may help elucidate underlying mechanisms and inform future therapeutic strategies.

## 2. Methods

A literature search was conducted using PubMed and Google Scholar between June and August 2026. Searches were performed using a combination of terms related to adipocytes or adipose tissue and incretin-based therapies. Incretin-based drugs were limited to GLP-1RAs and dual GIP/GLP-1RAs; other incretin-related therapies, such as dipeptidyl peptidase-4 inhibitors, were outside the scope of this review, as they indirectly modulate incretin signaling and can alter non-incretin peptide degradation [32]. Searches incorporated broad terms (GLP-1RA, incretin-based therapy) and specific treatments (including native GLP-1, native GIP, semaglutide, liraglutide, and tirzepatide). To identify additional relevant articles, citation searching of included primary studies and any review articles identified during the search was performed. The primary screening and study selection were conducted by J.K. Titles and abstracts were initially assessed for relevance, followed by full-text evaluation of potentially eligible articles.

Studies were eligible if they were in English and investigated the effects of a GLP-1RA or tirzepatide on adipose tissue (dWAT or sWAT), adipocytes (immature, mature, and during differentiation), adipocyte progenitors, or adipocyte-associated circulating mediators (adipokines or cytokines). Established and experimental incretin-based drugs were eligible. Studies involving cell lines, animal models, and human participants were included. Studies exclusively examining visceral adipose depots were excluded. When studies included multiple adipose depots, only data from dWAT or sWAT were retained when reported separately. Studies only reporting changes in body weight or fat mass, without an adipose-related outcome, were not included.

Data extraction and interpretation were performed by J.K. Extracted information included study characteristics, experimental model, adipocyte or adipose tissue source, intervention (drug, concentration, timing), and reported adipose-related outcomes. Particular attention was given to whether an intervention was administered before, during, or after adipocyte differentiation. Extracted data were organized into Supplementary Materials and findings were synthesized narratively according to the biological process affected.

## 3. Clinical Evidence Linking Incretin-Based Drugs and Hair Loss

Clinical data examining the potential association between incretin-based therapies and hair loss have accumulated from diverse sources varying in evidentiary strength, with strongest support provided by clinical trials (Supplementary Table S1) [5]. Although the specific type of hair loss is not always defined when reported as an adverse event, retrospective analyses have associated GLP-1RA use with adjusted odds ratios of 1.76 (95% Confidence Interval [CI]: 1.34–2.32) for TE and 1.64 (95% CI: 1.35–1.99) for AGA at 12-month follow-up [33]. Compared with other drug classes, GLP-1RA and GIP/GLP-1RA treatment was associated with higher alopecia risk than sodium-glucose cotransporter 2 inhibitors and dipeptidyl peptidase 4 inhibitors (Hazard Ratio [HR]: 1.37; 95% CI: 1.08–1.73 and HR: 1.68; 95% CI: 1.28–2.20, respectively), with corresponding event rates of 6.91 versus 5.04 and 6.53 versus 3.89 per 1000 patient years [34]. The association was even stronger for non-scarring alopecia specifically [34].

Semaglutide has been most extensively evaluated in relation to hair loss, likely reflecting its widespread use in clinical practice and resulting availability of adverse event data from clinical trials [35,36], retrospective studies [37,38], and disproportionality analyses [39,40]. In a clinical trial, alopecia occurred in 7% (23/334) of participants with obesity receiving semaglutide versus 3% (9/333) of those receiving placebo [36]. In retrospective analyses, reported HRs for alopecia risk in semaglutide users ranged from 2.08 (95% CI: 1.17–3.72; event rate: 26.5/1000 patient years; among women compared with bupropion-naltrexone, a non-GLP-1RA weight-loss drug) [37] to 4.43 (95% CI: 3.81–5.16; event number: 797/584,475 [0.14%]; among patients with diabetes compared with metformin) [38]. Notably, the association in the former analysis was not significant for men [37], suggesting a potential sex-specific difference in alopecia risk.

Other GLP-1RAs, such as liraglutide and exenatide, may also promote hair loss [6,38,41], although the available evidence remains limited, possibly reflecting their relatively lower utilization or more modest weight loss compared to other agents [5]. Ching et al. found that patients receiving liraglutide may have increased risk of alopecia, whereas treatment with exenatide may be associated with specific alopecia subtypes, namely TE and AGA [6]. Although these retrospective studies cannot establish causality and are subject to potential selection bias and confounding, the observation of hair loss across multiple GLP-1RAs provides an important signal warranting further prospective investigation.

Moreover, the dual GIP/GLP-1RA tirzepatide has also been associated with hair loss [8,38,40,42,43,44]. Across the SURMOUNT clinical trial program, alopecia occurred more frequently among tirzepatide-treated participants than placebo-treated controls (5.22% [99/1896] versus 0.93% [6/643] in SURMOUNT-1 and 6.97% [20/287] versus 1.37% [4/292] in SURMOUNT-3) [42,43]. In a retrospective analysis of patients with type 2 diabetes, tirzepatide use was associated with a higher risk of AGA (0.14% [273/197,335]) than semaglutide (HR: 1.53; 95% CI: 1.29–1.82) and liraglutide (HR: 2.50; 95% CI: 2.07–3.02) [38]. Compared with other treatments, reported hazard ratios ranged from 2.65 (95% CI: 1.32–5.35; versus various unrelated weight-loss drugs) [8] to 5.67 (95% CI: 4.42–7.28; versus metformin in patients with diabetes) [38].

Despite evidence suggesting a link between incretin-based therapies and hair loss, results have not been consistent between studies [39,45,46], indicating that the relationship between GLP-1RAs or dual GIP/GLP-1RAs and hair loss may vary across different clinical and treatment contexts. Nevertheless, the overall body of evidence supports hair loss as a clinically relevant adverse effect of incretin-based therapies. Indeed, hair loss has been identified as a reason for discontinuing GLP-1RA treatment [47], underscoring its negative impact on treatment adherence and patient quality of life.

## 4. Hair Follicle Biology and Cycling

The association between incretin-based drug use and non-scarring alopecias suggests that hair loss may arise from alterations in hair follicle homeostasis and disruption of follicular cycling dynamics rather than irreversible follicular destruction. This potential pathway is particularly relevant for TE, which results from premature transition of hair follicles from the growth phase into the resting phase. In contrast, AGA is characterized by progressive follicular miniaturization related to hormonal factors. Thus, the documented association with AGA may reflect accelerated clinical recognition or exacerbation of previously subclinical disease, although a direct biological effect cannot be excluded. Given these differences in pathobiology, further research will be required to determine whether distinct mechanisms contribute to different alopecia subtypes.

**Figure 1:**
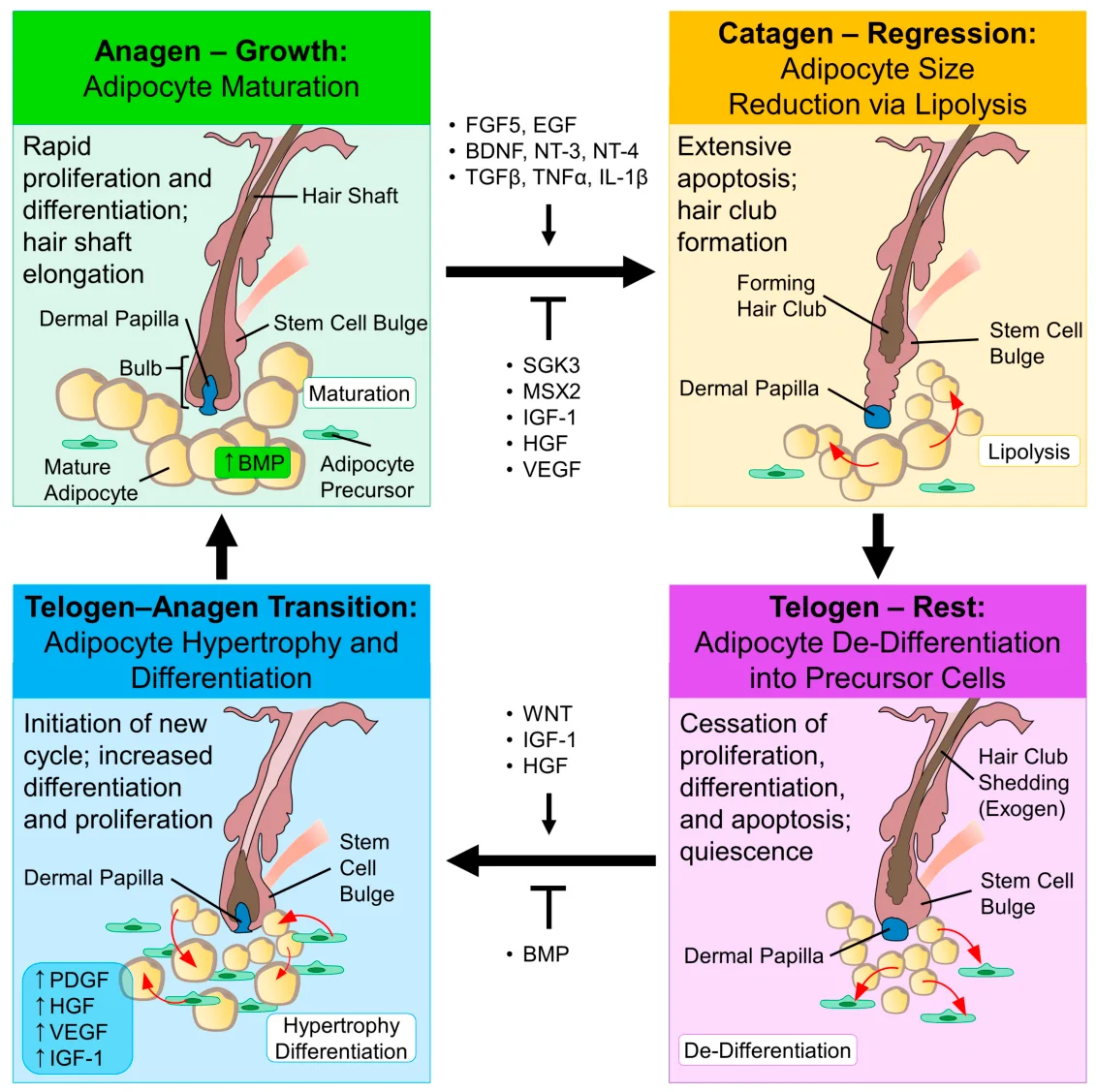
Schematic of Dermal Adipocyte Remodeling During Hair Follicle Cycling. The major phases of the hair follicle cycle are shown together with coordinated changes in the surrounding dermal white adipose tissue, which have been qualitatively illustrated based primarily on murine data [12,13,14,18,50,51]. In each panel, red arrows indicate adipocyte processes, as described in the white rectangles, while the colored rectangles indicate adipocyte-secreted mediators. During the transition into anagen, adipocyte precursors, including adipose-derived stem cells and preadipocytes, promote hair growth through the secretion of various growth factors, including platelet-derived growth factor (PDGF). As these precursors differentiate into mature adipocytes, the precursor pool and PDGF levels decline, whereas mature adipocytes expand through hypertrophy and increase production of bone morphogenetic proteins (BMPs). Throughout anagen, precursor-derived mediators maintain growth, whereas elevated BMP, along with specific growth factors, neurotrophins, and cytokines, contribute to catagen entry. In catagen, extensive apoptosis occurs within the lower portion of the hair follicle, accompanied by hair club formation. During the catagen-telogen transition, mature adipocytes undergo lipolysis and de-differentiation, resulting in dWAT regression and replenishment of the precursor population. The hair follicle enters a relatively quiescent state and may shed the hair club via exogen prior to the initiation of a new hair cycle. BDNF: Brain-Derived Neurotrophic Factor; BMP: Bone Morphogenetic Protein; EGF: Epidermal Growth Factor; FGF5: Fibroblast Growth Factor 5; HGF: Hepatocyte Growth Factor; IGF-1: Insulin-like Growth Factor-1; IL-1β: Interleukin-1 Beta; MSX2: Muscle Segment Homeobox 2; NT: Neurotrophin; PDGF: Platelet-Derived Growth Factor; SGK3: Serum and Glucocorticoid Responsive Kinase 3; TGFβ: Transforming Growth Factor Beta; TNFα: Tumor Necrosis Factor Alpha; VEGF: Vascular Endothelial Growth Factor.

Healthy hair follicles undergo continuous cycling through three traditional phases: anagen (growth), catagen (regression), and telogen (rest) [48,49]. A club hair forms during catagen and is retained through telogen before it may be shed through a distinct process termed exogen, which has been proposed as a fourth phase of the hair cycle [48,49]. Individual follicles cycle asynchronously, allowing hair growth to be maintained while different follicles occupy different stages of the cycle. This dynamic process is tightly managed by complex biologic signals, including interactions between follicular cells and dermal adipocytes in the surrounding dWAT (Figure 1) [13,14,48,49].

During the anagen phase, a subset of follicular stem cells within the bulge region undergo extensive proliferation and differentiation into distinct epithelial lineages to support follicular growth [14,48,49]. Sustained anagen growth is maintained by signaling factors such as serum and glucocorticoid responsive kinase 3 (SGK3), muscle segment homeobox 2 (MSX2), insulin-like growth factor-1 (IGF-1), hepatocyte growth factor (HGF), and vascular endothelial growth factor (VEGF) [48,49,50].

Following completion of anagen, hair follicles undergo a regulated transition into catagen, a brief regression phase defined by cessation of stem cell proliferation and increased apoptosis [48,49]. This transition is mediated by a shift toward catagen-promoting growth factors (fibroblast growth factor 5 [FGF5] and epidermal growth factor [EGF]), neurotrophins (brain-derived neurotrophic factor [BDNF], NT-3, NT-4), and cytokines (transforming growth factor beta-1 [TGFβ1], TNFα, and IL-1β) [48,49]. Hair follicles subsequently enter telogen, during which follicular cells exhibit minimal proliferation, differentiation, or apoptosis [48,49].

## 5. Dermal White Adipose Tissue Regulates Hair Follicle Cycling

Regulation of the hair cycle extends beyond the follicle itself and depends on paracrine signals from the closely associated dWAT, where complex interactions between follicular cells and dermal adipocytes primarily attached to the lower portion of the follicle can influence follicular cycling [12,13,14,15,16,17,48,49]. Dermal adipocytes originate from mesenchymal stromal cells (also referred to as adipose-derived stem cells [ADSCs]), which can differentiate into preadipocytes and subsequently mature adipocytes [13,14]. Mature adipocytes can also undergo de-differentiation into adipocyte-derived preadipocytes, allowing dynamic remodeling of the adipose compartment [12,13,14]. These changes closely parallel transitions in the hair follicle cycle, with substantial alterations in dWAT volume occurring throughout different phases [12,13,14,18].

The greatest expansion in dWAT occurs during early anagen, which is followed by progressive regression during catagen and telogen [12,13,14,18] (Figure 1). Immediately prior to anagen entry, approximately 50% of adipocyte precursors begin to proliferate, initiating a wave of adipogenic differentiation and hypertrophy that doubles dWAT thickness and increases adipocyte size [12,14]. Gradual depletion of the preadipocyte pool and continued accumulation of mature adipocytes alters the cellular composition of dWAT, inducing termination of the hair follicle growth phase [12,13,14]. During catagen, dermal adipocytes undergo de-differentiation and lipolysis rather than apoptosis, corresponding to a 50% reduction in dWAT volume by telogen [12,13,18]. These findings are also supported by human studies demonstrating a reduction in the size of hair follicle-associated dermal adipocytes during the anagen-to-catagen transition [50].

Animal models further demonstrate that dWAT actively regulates hair follicle cycling. Mice lacking myelin protein zero-like 3 (MPZL3), a protein involved in metabolism and energy expenditure, have reduced dWAT thickness and decreased dermal adipocyte size, accompanied by alopecia resulting from anagen dysregulation, highlighting the importance of the adipose niche for normal hair growth [52]. Moreover, early B-cell factor 1 (EBF1)-deficient mice lack preadipocytes and exhibit hair follicles arrested in late catagen or telogen, whereas administration of preadipocytes restores anagen re-entry [14]. Thus, dermal adipocytes comprise an adaptable tissue compartment that coordinates hair follicle cycling, with adipocyte precursors playing a particularly important role in anagen initiation.

The pro-anagen effects of dWAT are largely mediated through platelet-derived growth factor (PDGF), which is highly expressed in adipocyte precursors [14]. PDGF signaling stimulates follicular stem cells to promote the telogen-to-anagen transition, whereas loss of PDGF prevents anagen entry through impaired follicular cell activation [13,14]. In line with this, intradermal administration of PDGF-coated beads into *Ebf1*^−/−^ mice induces dose-dependent restoration of anagen [14], demonstrating that exogenous PDGF can compensate for the loss of adipocyte precursors. Additional precursor-derived factors, such as HGF, VEGF, and IGF-1, may also play a role in anagen maintenance [48,49,50,53]. In human hair follicle-dWAT cocultures, preadipocyte-secreted HGF stimulated proliferation of hair matrix keratinocytes and tended to promote hair shaft production [50].

In contrast, mature adipocytes predominantly promote follicular regression. As precursor differentiation increases the number of mature adipocytes during anagen, a concomitant enhancement in bone morphogenetic protein (BMP) signaling promotes transition to catagen [12,13,14]. In particular, BMP2 inhibits bulge stem cell activity, thus suppressing the follicular activation required for anagen maintenance [14,49,54]. Mature adipocytes can also promote inflammation through visfatin, resistin, and pro-inflammatory cytokines that further disturb normal hair cycling [11,55,56,57,58,59,60]. Although leptin and adiponectin have been implicated in hair growth [61,62], the contributions of these adipokines are not fully established. Notably, A-ZIP/F1 mice retain adipocyte precursors but lack mature adipocytes, yet maintain relatively normal follicular cycling kinetics [14]. As such, current evidence more strongly supports a catagen-inducing role for mature adipocytes [12,13,14].

Overall, the ratio of precursors and mature adipocytes within dWAT represents an important regulator of hair follicle cycling, and disruption of this balance may contribute to hair loss. However, the role of adipocytes in drug-mediated effects remains an area of active investigation, especially given the functional diversity of adipocyte populations. The evolving classification of adipocytes has contributed to considerable ambiguity in the interpretation of existing literature, as skin-associated adipocytes, now termed dWAT [17], have historically been referred to as subcutaneous WAT (sWAT) [63,64]. Moreover, the lack of cell type-specific markers and standardized approaches for isolating dWAT [12] complicates the distinction between these adipose depots. Accordingly, studies examining sWAT are included in this review while recognizing that these data may not exclusively reflect contributions from dWAT.

## 6. Systemic Consequences of Incretin-Based Therapies

Rapid weight loss among users of incretin-based therapies is associated with energy deficits and nutritional shifts arising from drug effects on appetite, satiety, and gastric emptying [1,2,10,65]. Reduced appetite and increased satiety can substantially decrease intake of calories, with reported caloric reductions of up to 39% [65]. Clinical trial data suggest that the magnitude of weight loss may be associated with a higher incidence of reported hair loss among incretin-based therapy users; however, consistent dose–response or drug-specific relationships have not been clearly demonstrated. For instance, hair loss incidence remained relatively similar between tirzepatide doses of 5, 10, and 15 mg [42]. Additionally, hair loss incidence was comparable across trials of different GLP-1RAs and GIP/GLP-1RAs, with reports in 2–7% of participants receiving semaglutide [35,36], 7% receiving liraglutide [41], and 4.9–7% receiving tirzepatide [42,43]. Nevertheless, a similar association with hair loss has been observed following bariatric surgery [8,66], suggesting that the physiological effects of weight reduction are important mediators of hair loss, although drug-specific effects could also play a role.

Given the high metabolic activity of hair follicles, especially during the anagen phase, caloric restriction may act as a stressor capable of disrupting the hair cycle and accelerating telogen entry [10]. Nutrient intake and absorption may also be reduced as a consequence of decreased food consumption and delayed gastric emptying [10]. To compensate for limited nutrient supply during incretin-based therapy, adipose tissue may undergo involution through increased lipolysis [24,25,67,68] and decreased hypertrophy [67,69,70]. As changes in adipose mass have been shown to parallel follicular cycling [12,13,14,18], acute reductions in adipose tissue may further contribute to early telogen onset. Moreover, deficiencies in micronutrients, including iron, zinc, and various vitamins, have also been associated with hair loss [10,71,72]. Collectively, these systemic changes in energy and nutrient availability are important potential contributors to hair cycle dysregulation and resulting hair loss as part of a multifactorial process.

## 7. Incretin-Based Therapies May Alter Adipocyte Differentiation in a Context-Dependent Manner

Besides systemic effects, incretin-based drugs also act directly on adipocytes, including modulation of adipocyte precursor differentiation. Importantly, none of the identified studies directly examined human dermal adipocytes. Supporting evidence is derived primarily from cellular studies (Supplementary Table S2) [19,20,21,68,73,74,75,76,77,78,79,80,81,82,83], most of which have used immature 3T3-L1 preadipocytes that may resemble dermal adipocytes [12]. In these murine cells, GLP-1 and liraglutide reliably promoted adipocyte differentiation, as demonstrated by increased expression of adipogenic transcription factors (peroxisome proliferator-activated receptor gamma [PPARγ] and CCAAT/enhancer-binding protein [C/EBP]), which initiate the adipocyte differentiation program, together with increased expression of mature adipocyte markers involved in lipogenesis, including lipoprotein lipase (LPL) and fatty acid-binding protein 4 (FABP4) [19,20,73,74,76,77].

In contrast, studies using human cells report less consistent findings. GLP-1 and liraglutide decreased expression of adipogenic transcription factors (PPARγ, C/EBPα, and adipocyte differentiation-related protein [ADRP]) and lipogenic genes (LPL and FABP4), while increasing lipolytic genes (hormone-sensitive lipase [HSL], perilipin, and patatin-like phospholipase domain containing-2 [PNPLA2]) [21,75,78,79,80]. However, these effects may depend on treatment timing, as pre-treatment of human mesenchymal stromal cells with a different GLP-1RA, exendin-4, enhanced adipogenic differentiation and increased PPARγ and FABP4 mRNA expression, whereas concurrent treatment produced the opposite effect [68].

**Figure 2:**
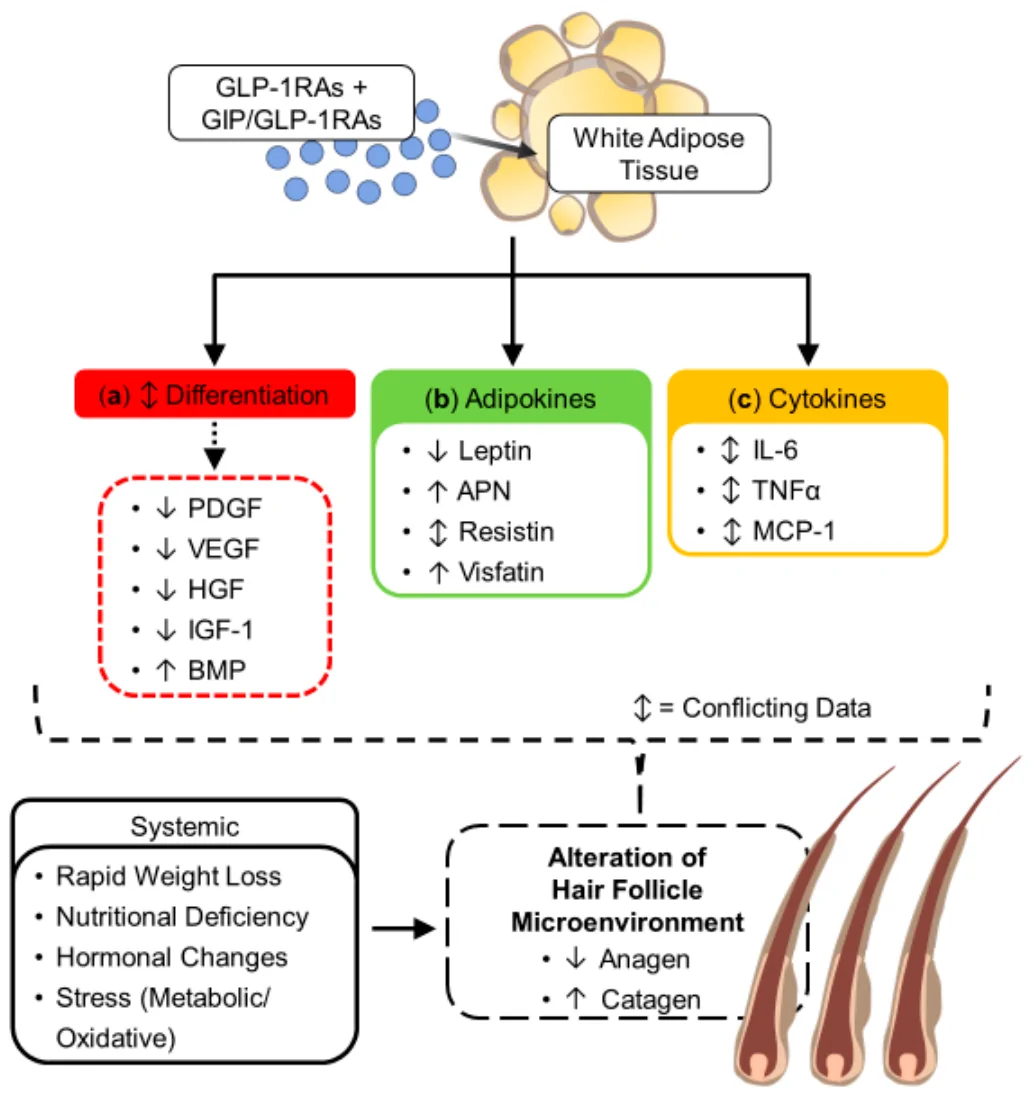
Proposed Mechanism of Adipocyte-Mediated Hair Loss Following Treatment with Incretin-Based Drugs. Preclinical data, mainly derived from cell lines or subcutaneous adipose tissue (solid colored boxes), suggest that GLP-1RAs and potentially GIP/GLP-1RAs may promote adipocyte reprogramming in a context-dependent manner by altering (**a**) differentiation (Section 7; red boxes), (**b**) adipokine secretion (Section 8; green box), and (**c**) cytokine production (Section 9; yellow box) [19,20,21,22,67,68,69,70,73,74,75,76,77,78,79,80,81,82,83,84,85,86,87,88,89,90,91,92,93,94,95,96,97,98,99,100,101,102,103]. Although not yet experimentally demonstrated (dotted boxes), drug-induced differentiation may reduce levels of precursor-derived growth factors involved in maintaining anagen while increasing the levels of proteins involved in catagen transition. Secreted adipokines may likewise be influenced by incretin-based treatments, including reductions in leptin that may prevent anagen initiation, and increases in adiponectin (APN) that may inhibit follicular growth. Other adipokines, together with direct effects of incretin-based drugs, may induce inflammation through increased production of specific cytokines. Together, this adipocyte reprogramming may alter the hair follicle microenvironment, resulting in premature anagen termination and early catagen onset. This hypothetical mechanism, although not yet adequately investigated, may partially explain the increased incidence of hair loss observed in patients receiving GLP-1RAs and GIP/GLP-1RAs. APN: adiponectin; BMP: bone morphogenetic protein; GIP: glucose-dependent insulinotropic polypeptide; GLP-1RA: glucagon-like peptide-1 receptor agonist; HGF: hepatocyte growth factor; IGF-1: insulin-like growth factor-1; IL-6: interleukin-6; MCP-1: monocyte chemoattractant protein-1; PDGF: platelet-derived growth factor; TNFα: tumor necrosis factor-alpha; VEGF: vascular endothelial growth factor; ↕: conflicting data across studies.

Alternatively, GIP and its analog (D-ALA^2^)-GIP both promoted adipocyte differentiation in murine cells [81,82,83]. However, the effects of tirzepatide on adipocyte differentiation have not been assessed in the included studies. Together, these preclinical data suggest that incretin signaling can regulate adipocyte differentiation, although the effects appear to be more context-dependent in human cells than in murine models (Figure 2a).

## 8. Incretin-Based Therapies May Modulate Adipokine Signaling

The drug-associated alterations in adipocyte differentiation may have several downstream effects, including changes in adipokine expression [53,104,105,106]. Several studies have demonstrated that incretin-based therapies can influence leptin and adiponectin expression in both humans and various experimental models.

A meta-analysis of 13 randomized controlled trials involving 1025 human participants demonstrated that GLP-1RAs significantly reduce serum leptin levels [107]. Although one human study found no change in circulating leptin following GLP-1RA treatment [89], in animal models of obesity, treatment with Boc5, exendin-4, liraglutide, semaglutide, and tirzepatide consistently reduced circulating leptin [69,70,84,86,87,91]. This finding is further supported by several preclinical studies where various incretin-based treatments commonly decreased leptin expression across *in vivo* models and *in vitro* adipocyte cultures (Supplementary Table S3) [69,70,84,85,86,87,88,89,90,91].

Complementary cell culture studies, including in ADSCs from human sWAT, further demonstrate that GLP-1RAs can directly reduce adipocyte-derived leptin [84,85,88]. GIP administration alone also decreased leptin mRNA expression in differentiated 3T3-L1 CAR adipocytes overexpressing the GIP receptor, suggesting that both incretin pathways may contribute to the downregulation of adipocyte-derived leptin [90]. No included cellular studies assessed leptin expression following treatment with tirzepatide. Collectively, these preliminary findings indicate that incretin-based therapies may act on adipocytes to suppress leptin production (Figure 2b).

Adiponectin is another adipokine that has been shown to be altered following treatment with GLP-1RAs and GIP/GLP-1RAs. A meta-analysis assessing 1497 participants from 20 randomized controlled trials demonstrated that GLP-1RAs increase circulating adiponectin levels, with liraglutide showing the greatest effect [108]. Additional evidence from clinical studies, animal models, and adipose tissue-based experiments also indicates that these agents generally increase adiponectin mRNA expression and protein levels, although responses vary depending on the experimental model, adipocyte differentiation state, and specific therapy evaluated (Supplementary Table S4) [67,69,70,75,77,78,86,87,88,89,90,91,92,93,94,95,96,97]. Circulating adiponectin typically increased following treatment with various GLP-1RAs or tirzepatide [69,70,91,95,96,97], although decreases [86] and no significant changes [87,89] have also been reported.

At the adipose tissue level, GLP-1 administration did not change adiponectin mRNA expression in sWAT explants [75], whereas exendin-4 increased adiponectin in murine adipose tissue [94]. In isolated adipocytes, adiponectin production following GLP-1RA treatment was variably affected [67,75,92], although many studies demonstrated an increase following treatment with exendin-4 [93,94] or liraglutide [77,78,88]. Evidence for GIP-mediated regulation of adiponectin remains limited, with a single murine cell-line study reporting decreased adiponectin mRNA expression [90]. To our knowledge, tirzepatide has not been specifically assessed for effects on adiponectin in isolated adipocytes. Taken together, GLP-1RAs appear to predominantly elevate adiponectin production in adipocytes (Figure 2b).

Other adipokines, such as resistin and visfatin, have been comparatively understudied in the context of GLP-1RAs and GIP/GLP-1RAs, with reports indicating variable effects (Supplementary Table S5, Figure 2b) [22,69,86,87,89,91,92,98]. Most studies have measured circulating resistin [69,86,87,89,91], with only a single study examining resistin secretion directly from adipocytes and reporting increased resistin protein levels following GIP treatment [98]. In addition, available evidence indicates that liraglutide may increase circulating visfatin in humans [89], whereas GLP-1 or exendin-4 treatment increases visfatin expression in murine and human adipocyte cell lines [22,92]. Future studies should evaluate clinically relevant incretin-based therapies using translational animal models and human participants to determine the relevance of these observations.

## 9. Incretin-Based Therapies May Shift Inflammatory Signaling

Beyond adipokines, adipocytes secrete pro-inflammatory cytokines, a function that may also be affected by drug treatment. A recent meta-analysis examining 1762 participants with type 2 diabetes found moderate to high heterogeneity across clinical studies assessing circulating IL-6, TNFα, and monocyte chemoattractant protein-1 (MCP-1) levels following treatment with GLP-1RAs and GIP/GLP-1RAs [109]. Similar variability is also observed in adipocyte-focused studies (Supplementary Table S6) [69,75,77,85,86,88,89,90,91,93,99,100,101,102,103].

Heterogeneity in reports assessing cytokine expression appears to depend on both the experimental model and the specific incretin-based drug studied. The limited number of studies available for individual drugs further restricts interpretation of these findings. While GLP-1RAs produced inconsistent changes in IL-6, TNFα, and MCP-1 expression [69,75,77,85,86,88,89,93,99,100], GIP typically increased these inflammatory mediators in adipocytes, although responses may depend on the specific cell source [90,101,102,103]. No included tirzepatide studies assessed cell-specific cytokine production.

Overall, these preliminary findings highlight considerable uncertainty regarding the effects of incretin-based therapies on adipocyte-derived cytokine production (Figure 2c). Although incretin-based drugs are thought to exert anti-inflammatory effects through modulation of immune cell signaling and reduction in reactive oxygen species [110], these effects may be specific to certain cell types or tissues, highlighting the need for further mechanistic and translational studies.

## 10. Proposed Mechanistic Model of Drug-Induced Hair Loss

Overall, we propose that drug-induced remodeling of WAT may represent one of several pathways that contribute to hair loss by changing adipocyte-derived signals that regulate follicular homeostasis (Figure 2). This hypothesis is primarily supported by data involving GLP-1RAs, as evidence for direct effects of GIP/GLP-1RAs at the adipocyte level remains limited. This potential mechanism may have particular relevance for TE; however, it is important to note that the proposed model remains hypothetical, as no studies to date have directly established that GLP-1RAs or GIP/GLP-1RAs alter human dWAT, or that such changes contribute to the development of TE or AGA.

Nevertheless, under physiological conditions, adipocyte differentiation is synchronized with hair follicle cycling. As GLP-1RAs appear to promote differentiation in specific contexts, these drugs may disrupt the temporal coordination of dWAT remodeling and alter the ratio of immature and mature adipocyte populations required for follicular homeostasis. Indeed, a recent prospective study by Firsowicz et al. found a ~4-fold decrease in ADSCs in sWAT from female participants (aged 25–65 years) with abdominal skin laxity receiving semaglutide or tirzepatide (exposure duration: 12–27 months) compared with untreated controls [111]. Although these findings require validation in scalp dWAT and larger study populations, the depletion of ADSCs and immature preadipocytes, coupled with an increase in mature adipocytes, could contribute to hair loss, possibly through alteration of the PDGF/BMP ratio [13,14]. This shift may shorten anagen and/or prolong telogen, potentially contributing to the development of non-scarring alopecias, especially TE. Studies of human scalp tissue are needed to determine whether such changes occur following treatment, as these effects currently represent conceptual extrapolations rather than directly measured biomarkers.

In addition to altering adipocyte population dynamics, potential changes in differentiation may also influence the adipocyte secretome [53,104,105,106], possibly affecting hair follicle cycling. For instance, drug-associated increases in adiponectin may negatively regulate HGF signaling through downregulation of its receptor, c-MET, potentially limiting hair follicle growth [112]. Suppression of leptin may also impair hair growth [61], while resistin and visfatin can induce inflammation [55,57], which is further amplified by cytokines. IL-6 and TNFα may disrupt hair cycling by reducing hair shaft elongation and promoting catagen entry [58] or follicular dystrophy [59], whereas MCP-1 may increase macrophage infiltration into adipose tissue [60]. As IL-6 and TNFα appear to be elevated in AGA [113,114], their modulation by GLP-1RAs may contribute to alopecia. Collectively, these preliminary findings support a hypothetical model in which treatment may influence hair follicle cycling through direct effects on adipocytes, including induction of adipogenic differentiation and changes in secretory function. These data provide a rationale for future mechanistic studies using human dWAT models to evaluate whether drug-induced adipocyte changes indeed mediate alterations in hair follicle cycling.

## 11. Limitations of Current Literature

This review synthesizes the available literature supporting the direct effects of GLP-1RAs and GIP/GLP-1RAs on adipocyte biology; however, current evidence is primarily derived from adipocyte cell lines and studies examining sWAT in animal models. Circulating adipokine and cytokine measurements cannot be interpreted as evidence of adipocyte-specific alteration, as these mediators may originate from multiple cell types. For instance, resistin is abundantly produced by murine adipocytes, whereas in humans, it is produced primarily by monocytes and macrophages [115]. Similarly, it is difficult to distinguish drug-specific effects from those secondary to weight loss and systemic metabolic changes. Given that weight loss of approximately 15% alone may trigger TE regardless of its underlying cause [116], studies using active comparators with similar degrees of weight reduction are needed to better isolate pharmacological effects. Moreover, to our knowledge, no studies have yet examined the effects of GLP-1RAs or GIP/GLP-1RAs on isolated dermal adipocyte populations or within hair follicles. Consequently, reliance on preclinical models limits the extent to which data can be extrapolated to human dWAT, highlighting the need for mechanistic studies in patients developing drug-associated alopecia.

Although sWAT and dWAT represent distinct adipose depots, their shared characteristics suggest some functional overlap, allowing sWAT findings to provide a useful foundation for generating hypotheses that should be subsequently validated in dWAT. Prior to the recognition of dWAT as a distinct entity, Walker et al. distinguished between “superficial” and “deep” layers of sWAT in biopsies from healthy human participants [117]. No differences in adiponectin or TNFα secretion were observed between these layers; however, superficial sWAT exhibited lower resistin mRNA expression and higher leptin secretion compared with deeper sWAT [117]. More recently, Zhang et al. established a protocol to purify mature dermal adipocytes from mice and compared these cells with isolated subcutaneous adipocytes using RNA sequencing, demonstrating comparable expression of canonical adipocyte markers, including PPARγ, FABP4, leptin, and adiponectin [12]. However, dWAT also exhibited depot-specific features, including marked enrichment of cathelicidin antimicrobial peptide (CAMP), as well as differences in browning-related pathways and metabolite production [12]. Despite shared fundamental adipocyte characteristics, the specialized functions of different adipose depots underscore the need to replicate findings in appropriate tissue contexts to characterize drug-induced effects.

Evolving adipocyte classification and uncertainty regarding GLP-1 receptor and GIP receptor expression have further complicated interpretation of current data. The fibroblast 3T3-L1 cell line, derived from a mouse embryo, remains the principal *in vitro* model for adipocyte research; however, the mature adipocyte phenotype represented by this model remains uncertain. Zhang et al. proposed that these cells may resemble dermal adipocytes, based on their high expression of CAMP, although the lack of cell type-specific markers continues to limit classification [12]. Additionally, GLP-1 receptor and GIP receptor expression may be dependent on the specific cells assessed and their differentiation state. Although GLP-1 receptor expression has been reported in 3T3-L1 cells [22,23] and supported by studies using receptor knockdown [19] and receptor antagonism [20], evidence in human cells is more heterogeneous, with expression detected in human ADSCs and differentiated adipocytes in some studies [21,24], but not in others [25]. GIP receptor expression appears to be restricted to differentiated adipocytes [26,27,29,30,31], with the strongest human evidence derived from studies utilizing RNAScope [25] or single-nucleus RNA sequencing [28]. However, these studies examined subcutaneous adipocytes and require confirmation in dermal adipocytes.

Consequently, findings from available adipocyte models must be interpreted as hypothesis-generating evidence for potential drug-mediated regulation of adipocyte function. Future studies are required to independently determine the relative contributions of GLP-1 receptor and GIP receptor activation, and to establish whether these effects extend specifically to dWAT within the hair follicle niche.

## 12. Knowledge Gaps and Future Directions

Adipocytes represent only one component of the complex hair follicle microenvironment, and the effects of GLP-1RAs and GIP/GLP-1RAs within this region are not well characterized. These drugs have been shown to promote browning of white adipocytes, resulting in the development of a “beige” phenotype with increased thermogenic capacity [69,118,119,120,121]. However, the consequences of this phenotypic shift for follicular cycling remain unknown. Moreover, interactions with additional cell populations, including immune and other dermal cells, may also contribute to treatment-associated hair loss. Systemic changes associated with incretin-based therapies, including changes in circulating glucose, cholesterol, and free fatty acid concentrations, may further influence energy availability and metabolic signaling in cells within hair follicles. Therefore, adipocyte reprogramming may contribute to drug-associated hair loss as part of a multifactorial mechanism involving both local and systemic alterations. Clinicians should remain aware of this potential adverse effect when prescribing incretin-based drugs.

As drug-associated hair loss risk may be elevated in females [5,37], sex-specific differences require systematic evaluation. Given the association with AGA [5], hormone signaling should be further investigated, particularly as one study suggests interactions between estrogen and GLP-1RAs in adipose tissue [122]. Sex differences in adipose distribution should also be considered, as women generally have greater sWAT and less visceral adipose tissue than men [123], although whether similar differences exist in scalp dWAT remains understudied. Likewise, sex-specific differences in hair cycling, including in telogen length and regrowth kinetics [124,125], require investigation for their potential relevance to hair loss mechanisms.

Further research is needed to better define specific clinical subtypes of hair loss and identify susceptible patient populations. Potential mechanisms require validation in appropriate tissue. Following confirmation of GLP-1 receptor and GIP receptor expression in human scalp dWAT, matched coculture experiments of hair follicles and adipocytes under clinically relevant free-drug concentrations should be conducted to assess hair-shaft elongation and adipocyte biology, including proliferation, differentiation, apoptosis, and secretion of applicable mediators. Studies should establish GLP-1 receptor and GIP receptor dependence through selective antagonism or genetic disruption, followed by rescue experiments using PDGF/HGF administration or BMP inhibition. Pair-fed or weight-matched animal models, as well as conditioned-medium experiments, would also help limit confounding and isolate drug-specific effects.

Nevertheless, elucidating adipocyte-mediated mechanisms may identify opportunities to prevent or mitigate hair loss in patients receiving incretin-based therapies. In particular, restoring adipocyte precursor-derived signaling may represent a promising exploratory research direction. Delivery of these signaling molecules, either through the topical application of ADSC-derived conditioned media (ADSC-CM) or via microneedle-mediated delivery of ADSC-derived exosomes, may promote hair growth, with early preclinical and clinical evidence in TE and AGA supporting their potential benefits [126,127,128]. The therapeutic potential of these mediators, as well as exogenous PDGF or HGF [14,50], warrants investigation to determine their efficacy for drug-associated hair loss, with key considerations including composition, dosing, tissue penetration, safety, and feasibility. By restoring signaling pathways disrupted through adipocyte precursor depletion, these approaches may help maintain normal hair follicle cycling in patients experiencing hair loss during incretin-based drug treatment.

## 13. Conclusions

This review examines the available evidence supporting adipocyte reprogramming as a hypothetical, non-exclusive, mechanistic link between incretin-based drugs and hair loss. Drug-associated alterations in adipocyte differentiation, adipokine secretion, and cytokine production have been variably demonstrated in preclinical adipocyte studies. If confirmed in dermal adipocytes within the scalp, it is possible that these changes may disrupt the hair follicle microenvironment and impair the adipocyte–follicle interactions required for normal hair cycling. A better understanding of these mechanisms in relevant dWAT models may facilitate the development of strategies that preserve hair follicle homeostasis while maintaining the substantial metabolic benefits of incretin-based drugs.

## Data Availability

No new data were created or analyzed in this study. Data sharing is not applicable to this article.

## Supplementary Materials

The following supporting information can be downloaded at https://www.mdpi.com/article/10.3390/biology15181642/s1, Supplementary Table S1: Clinical studies examining hair loss in users of incretin-based therapies, Supplementary Table S2: Effects of incretin-based treatments on adipogenic differentiation; Supplementary Table S3: Effect of incretin-based treatments on leptin; Supplementary Table S4: Effect of incretin-based treatments on adiponectin; Supplementary Table S5: Effects of incretin-based treatments on resistin and visfatin; Supplementary Table S6: Effects of incretin-based treatments on inflammatory cytokines.

## Abbreviations

The following abbreviations are used in this manuscript:

ADRP	Adipocyte Differentiation-Related Protein
ADSC	Adipose-Derived Stem Cell
ADSC-CM	Adipose-Derived Stem Cell Conditioned Media
AGA	Androgenetic Alopecia
APN	Adiponectin
BDNF	Brain-Derived Neurotrophic Factor
BMP	Bone Morphogenetic Protein
CAMP	Cathelicidin Antimicrobial Peptide
C/EBP	CCAAT/Enhancer-Binding Protein
CI	Confidence Interval
dWAT	Dermal White Adipose Tissue
EBF1	Early B-cell Factor 1
EGF	Epidermal Growth Factor
FABP4	Fatty Acid-Binding Protein 4
FGF5	Fibroblast Growth Factor 5
GIP	Glucose-dependent Insulinotropic Polypeptide
GLP-1RA	Glucagon-Like Peptide-1 Receptor Agonist
HGF	Hepatocyte Growth Factor
HR	Hazard Ratio
HSL	Hormone-Sensitive Lipase
IGF-1	Insulin-like Growth Factor-1
IL	Interleukin
LPL	Lipoprotein Lipase
MCP-1	Monocyte Chemoattractant Protein-1
MPZL3	Myelin Protein Zero-Like 3
MSX2	Muscle Segment Homeobox 2
NT	Neurotrophin
PDGF	Platelet-Derived Growth Factor
PNPLA2	Patatin-like Phospholipase Domain Containing 2
PPARγ	Peroxisome Proliferator-Activated Receptor Gamma
SGK3	Serum and Glucocorticoid Responsive Kinase 3
sWAT	Subcutaneous White Adipose Tissue
TE	Telogen Effluvium
TGFβ	Transforming Growth Factor Beta
TNFα	Tumor Necrosis Factor Alpha
VEGF	Vascular Endothelial Growth Factor
WAT	White Adipose Tissue

## References

[1] Muzurović E.M., Volčanšek Š., Tomšić K.Z., Janež A., Mikhailidis D.P., Rizzo M., Mantzoros C.S. (2022). Glucagon-like peptide-1 receptor agonists and dual glucose-dependent insulinotropic polypeptide/glucagon-like peptide-1 receptor agonists in the treatment of obesity/metabolic syndrome, prediabetes/diabetes and non-alcoholic fatty liver disease-current evidence. J. Cardiovasc. Pharmacol. Ther..

[2] Hamed K., Alosaimi M.N., Ali B.A., Alghamdi A., Alkhashi T., Alkhaldi S.S., Altowarqi N.A., Alzahrani H., Alshehri A.M., Alkhaldi R.K. (2024). Glucagon-like peptide-1 (GLP-1) receptor agonists: Exploring their impact on diabetes, obesity, and cardiovascular health through a comprehensive literature review. Cureus.

[3] Yeo Y.H., Rezaie A., Hsieh T.Y., Hu X., Gaddam S., Ma K.S., Mohamed G., Lee G.Y., Huang P., Watson R. (2024). Shifting trends in the indication of glucagon-like peptide-1 receptor agonist prescriptions: A nationwide analysis. Ann. Intern. Med..

[4] Gratzl S., Farrar K.G., Holler E., Vachon M.S., Masters N., Cartwright B.M.G. (2025). Monitoring report: GLP-1 RA prescribing trends—March 2026 data. medRxiv.

[5] Gupta A.K., Teasell E.M., Economopoulos V., Mirmirani P. (2026). GLP-1 therapies and hair loss: A systematic review of current evidence and implications for counseling. Sci. Prog..

[6] Ching L.M., Guirguis C.A., Tung J.K. (2025). Associations between GLP-1 receptor agonists and alopecia: A multi-centre retrospective analysis and public interest trends. JEADV Clin. Pract..

[7] Yu C., Xanthavanij N., Wang T.H., See X.Y., Chang Y., Chiang C., Hsia Y.P., Chang C., Yang K., Chiang C. (2025). Impact of glucagon-like peptide-1 receptor agonists on cutaneous events: A systematic review and meta-analysis. JAAD Rev..

[8] Neubauer Z., Ong M.M., Singal A., Lipner S.R. (2025). Increased risk of telogen effluvium with tirzepatide compared to other weight loss medications: A retrospective cohort TriNetX database study. J. Am. Acad. Dermatol..

[9] Urbina J., Salinas-Ruiz L.E., Valenciano C., Clapp B. (2026). Micronutrient and nutritional deficiencies associated with GLP-1 receptor agonist therapy: A narrative review. Clin. Obes..

[10] Spindler A., Maas D., Rachko G., Zappi I., Kapur S., Sidhu K., Maas K., Garza-García L., Patrocínio J., Petukhova L. (2026). Glucagon-like peptide-1 receptor agonists and hair loss: Current evidence and potential mechanisms. JAAD Rev..

[11] Marcinkowska A., Marcinkowska J., Jóźwiak S., Pawelec K. (2026). Adipose tissue as an endocrine organ: Updated insights into hormonal dysfunction in obesity. Int. Med. J..

[12] Zhang Z., Shao M., Hepler C., Zi Z., Zhao S., An Y.A., Zhu Y., Ghaben A.L., Wang M.-Y., Li N. (2019). Dermal adipose tissue has high plasticity and undergoes reversible dedifferentiation in mice. J. Clin. Investig..

[13] Kruglikov I.L., Zhang Z., Scherer P.E. (2019). The role of immature and mature adipocytes in hair cycling. Trends Endocrinol. Metab..

[14] Festa E., Fretz J., Berry R., Schmidt B., Rodeheffer M., Horowitz M., Horsley V. (2011). Adipocyte lineage cells contribute to the skin stem cell niche to drive hair cycling. Cell.

[15] Kruglikov I.L., Scherer P.E. (2016). Dermal adipocytes and hair cycling: Is spatial heterogeneity a characteristic feature of the dermal adipose tissue depot?. Exp. Dermatol..

[16] Kruglikov I.L., Scherer P.E. (2016). Dermal adipocytes: From irrelevance to metabolic targets?. Trends Endocrinol. Metab..

[17] Driskell R.R., Jahoda C.A.B., Chuong C.-M., Watt F.M., Horsley V. (2014). Defining dermal adipose tissue. Exp. Dermatol..

[18] Foster A.R., Nicu C., Schneider M.R., Hinde E., Paus R. (2018). Dermal white adipose tissue undergoes major morphological changes during the spontaneous and induced murine hair follicle cycling: A reappraisal. Arch. Dermatol. Res..

[19] Challa T.D., Beaton N., Arnold M., Rudofsky G., Langhans W., Wolfrum C. (2012). Regulation of adipocyte formation by GLP-1/GLP-1R signaling. J. Biol. Chem..

[20] Chen J., Zhao H., Ma X., Zhang Y., Lu S., Wang Y., Zong C., Qin D., Wang Y., Yang Y.Y. (2017). GLP-1/GLP-1R signaling in regulation of adipocyte differentiation and lipogenesis. Cell Physiol. Biochem..

[21] Lee H.M., Joo B.S., Lee C.H., Kim H.Y., Ock J.H., Lee Y.S. (2015). Effect of glucagon-like peptide-1 on the differentiation of adipose-derived stem cells into osteoblasts and adipocytes. J. Menopausal Med..

[22] Liu R., Ding X., Wang Y., Wang M., Peng Y. (2013). Glucagon-like peptide-1 upregulates visfatin expression in 3T3-L1 adipocytes. Horm. Metab. Res..

[23] Egan J.M., Montrose-Rafizadeh C., Wang Y., Bernier M., Roth J. (1994). Glucagon-like peptide-1(7-36) amide (GLP-1) enhances insulin-stimulated glucose metabolism in 3T3-L1 adipocytes: One of several potential extrapancreatic sites of GLP-1 action. Endocrinology.

[24] Vendrell J., El Bekay R., Peral B., García-Fuentes E., Megia A., Macias-Gonzalez M., Real J.F., Jimenez-Gomez Y., Escoté X., Pachón G. (2011). Study of the potential association of adipose tissue GLP-1 receptor with obesity and insulin resistance. Endocrinology.

[25] Regmi A., Aihara E., Christe M.E., Varga G., Beyer T.P., Ruan X., Beebe E., O’Farrell L.S., Bellinger M.A., Austin A.K. (2024). Tirzepatide modulates the regulation of adipocyte nutrient metabolism through long-acting activation of the GIP receptor. Cell Metab..

[26] Yip R.G., Boylan M.O., Kieffer T.J., Wolfe M.M. (1998). Functional GIP receptors are present on adipocytes. Endocrinology.

[27] McIntosh C.H., Bremsak I., Lynn F.C., Gill R., Hinke S.A., Gelling R., Nian C., McKnight G., Jaspers S., Pederson R.A. (1999). Glucose-dependent insulinotropic polypeptide stimulation of lipolysis in differentiated 3T3-L1 cells: Wortmannin-sensitive inhibition by insulin. Endocrinology.

[28] Yu X., Chen S., Funcke J.-B., Straub L.G., Pirro V., Emont M.P., Droz B.A., Collins K.A., Joung C., Pearson M.J. (2025). The GIP receptor activates futile calcium cycling in white adipose tissue to increase energy expenditure and drive weight loss in mice. Cell Metab..

[29] Ahlqvist E., Osmark P., Kuulasmaa T., Pilgaard K., Omar B., Brøns C., Kotova O., Zetterqvist A.V., Stancáková A., Jonsson A. (2013). Link between GIP and osteopontin in adipose tissue and insulin resistance. Diabetes.

[30] Kim S.-J., Nian C., McIntosh C.H.S. (2011). Adipocyte expression of the glucose-dependent insulinotropic polypeptide receptor involves gene regulation by PPARγ and histone acetylation. J. Lipid Res..

[31] Weaver R.E., Donnelly D., Wabitsch M., Grant P.J., Balmforth A.J. (2008). Functional expression of glucose-dependent insulinotropic polypeptide receptors is coupled to differentiation in a human adipocyte model. Int. J. Obes..

[32] Aertgeerts K., Ye S., Tennant M.G., Kraus M.L., Rogers J., Sang B.-C., Skene R.J., Webb D.R., Prasad G.S. (2004). Crystal structure of human dipeptidyl peptidase IV in complex with a decapeptide reveals details on substrate specificity and tetrahedral intermediate formation. Protein Sci..

[33] Vidal S.I., Akiska Y.M., Nasseri M., Menta N., Nussbaum D., Cotton C.H., Castelo-Soccio L., Friedman A. (2026). Increased risk of hair loss with GLP-1 receptor agonists: A real-world multicenter TrinetX cohort study. JAAD Int..

[34] Tang H., Zhang B., Lu Y., Zhang D., Liu R., Lu Y., Cotsarelis G., Asch D.A., Chen Y. (2026). Risk of hair loss associated with glucagon-like peptide-1 receptor agonists in adults with type 2 diabetes: Target trial emulation. BMJ.

[35] Eckard A.R., Wu Q., Sattar A., Ansari-Gilani K., Labbato D., Foster T., Fletcher A.A., Adekunle R.O., McComsey G.A. (2024). Once-weekly semaglutide in people with HIV-associated lipohypertrophy: A randomised, double-blind, placebo-controlled phase 2b single-centre clinical trial. Lancet Diabetes Endocrinol..

[36] Knop F.K., Aroda V.R., do Vale R.D., Holst-Hansen T., Laursen P.N., Rosenstock J., Rubino D.M., Garvey W.T. (2023). OASIS 1 Investigators. Oral semaglutide 50 mg taken once per day in adults with overweight or obesity (OASIS 1): A randomised, double-blind, placebo-controlled, phase 3 trial. Lancet.

[37] Sodhi M., Rezaeianzadeh R., Kezouh A., Frey C., Etminan M. (2025). Risk of Hair Loss with Semaglutide for Weight Loss. medRxiv.

[38] Singal A., Ong M.M., Neubauer Z., Lipner S.R. (2025). Association between androgenetic alopecia and glucagon-like peptide-1 receptor treatment in type 2 diabetes mellitus patients: A retrospective cohort study. Int. J. Dermatol..

[39] Kim T.H., Lee K., Park S., Oh J., Park J., Jo H., Lee H., Cho J., Wen X., Cho H. (2025). Adverse drug reaction patterns of GLP-1 receptor agonists approved for obesity treatment: Disproportionality analysis from global pharmacovigilance database. Diabetes Obes. Metab..

[40] Godfrey H., Leibovit-Reiben Z., Jedlowski P., Thiede R. (2025). Alopecia associated with the use of semaglutide and tirzepatide: A disproportionality analysis using the FDA adverse event reporting system (FAERS) from 2022 to 2023. J. Eur. Acad. Dermatol. Venereol..

[41] Danne T., Biester T., Kapitzke K., Jacobsen S.H., Jacobsen L.V., Petri K.C.C., Hale P.M., Kordonouri O. (2017). Liraglutide in an adolescent population with obesity: A randomized, double-blind, placebo-controlled 5-week trial to assess safety, tolerability, and pharmacokinetics of liraglutide in adolescents aged 12–17 years. J. Pediatr..

[42] Jastreboff A.M., Aronne L.J., Ahmad N.N., Wharton S., Connery L., Alves B., Kiyosue A., Zhang S., Liu B., Bunck M.C. (2022). Tirzepatide once weekly for the treatment of obesity. N. Engl. J. Med..

[43] Wadden T.A., Chao A.M., Machineni S., Kushner R., Ard J., Srivastava G., Halpern B., Zhang S., Chen J., Bunck M.C. (2023). Tirzepatide after intensive lifestyle intervention in adults with overweight or obesity: The SURMOUNT-3 phase 3 trial. Nat. Med..

[44] Carmichael C., Jouravskaya I., Collins E., Burns D., Poon J.L., Kitchen H., Mojdami D., Murphy M., Ahmad N., Kanu C. (2025). Patient experience of treatment with tirzepatide for weight management: Exit interviews from SURMOUNT-4. Patient.

[45] Jairath R., Abreu D., Mann C. (2024). 53028 Semaglutide and hair loss: A real-world analysis. J. Am. Acad. Dermatol..

[46] Gong A.H., Sayegh J., Garcia D., Ghanian S. (2025). 0224 Dermatologic outcomes of patients taking semaglutide: A propensity-matched large cohort study. J. Investig. Dermatol..

[47] Liu S., Wong G., Mavi P., Gysel S.C., Burton D., Monteiro N., Durocher D., Sharma A.M., Tsuyuki R.T. (2026). Factors contributing to non-persistence of glucagon-like peptide-1 agonists: A cross-sectional study. Obes. Med..

[48] Alonso L., Fuchs E. (2006). The hair cycle. J. Cell Sci..

[49] Schneider M.R., Schmidt-Ullrich R., Paus R. (2009). The hair follicle as a dynamic miniorgan. Curr. Biol..

[50] Nicu C., O’Sullivan J.D.B., Ramos R., Timperi L., Lai T., Farjo N., Farjo B., Pople J., Bhogal R., Hardman J.A. (2021). Dermal adipose tissue secretes HGF to promote human hair growth and pigmentation. J. Investig. Dermatol..

[51] Yong A.A., Unger R., Shapiro R. (2023). Hair follicle physiology and mechanisms of hair disorders. Hair Transplantation.

[52] Leiva A.G., Chen A.L., Devarajan P., Chen Z., Damanpour S., Hall J.A., Bianco A.C., Li J., Badiavas E.V., Zaias J. (2014). Loss of Mpzl3 function causes various skin abnormalities and greatly reduced adipose depots. J. Investig. Dermatol..

[53] Salgado A.J.B.O.G., Reis R.L.G., Sousa N.J.C., Gimble J.M. (2010). Adipose tissue derived stem cells secretome: Soluble factors and their roles in regenerative medicine. Curr. Stem Cell Res. Ther..

[54] Plikus M.V., Mayer J.A., de la Cruz D., Baker R.E., Maini P.K., Maxson R., Chuong C.-M. (2008). Cyclic dermal BMP signalling regulates stem cell activation during hair regeneration. Nature.

[55] Fu Y., Luo L., Luo N., Garvey W.T. (2006). Proinflammatory cytokine production and insulin sensitivity regulated by overexpression of resistin in 3T3-L1 adipocytes. Nutr. Metab..

[56] Moschen A.R., Kaser A., Enrich B., Mosheimer B., Theurl M., Niederegger H., Tilg H. (2007). Visfatin, an adipocytokine with proinflammatory and immunomodulating properties. J. Immunol..

[57] Liu S.W., Qiao S.B., Yuan J.S., Liu D.Q. (2009). Visfatin stimulates production of monocyte chemotactic protein-1 and interleukin-6 in human vein umbilical endothelial cells. Horm. Metab. Res..

[58] Kwack M.H., Ahn J.S., Kim M.K., Kim J.C., Sung Y.K. (2012). Dihydrotestosterone-inducible IL-6 inhibits elongation of human hair shafts by suppressing matrix cell proliferation and promotes regression of hair follicles in mice. J. Investig. Dermatol..

[59] Philpott M.P., Sanders D.A., Bowen J., Kealey T. (1996). Effects of interleukins, colony-stimulating factor and tumour necrosis factor on human hair follicle growth in vitro: A possible role for interleukin-1 and tumour necrosis factor-alpha in alopecia areata. Br. J. Dermatol..

[60] Kanda H., Tateya S., Tamori Y., Kotani K., Hiasa K., Kitazawa R., Kitazawa S., Miyachi H., Maeda S., Egashira K. (2006). MCP-1 contributes to macrophage infiltration into adipose tissue, insulin resistance, and hepatic steatosis in obesity. J. Clin. Investig..

[61] Sumikawa Y., Inui S., Nakajima T., Itami S. (2014). Hair cycle control by leptin as a new anagen inducer. Exp. Dermatol..

[62] Won C.H., Yoo H.G., Park K.Y., Shin S.H., Park W.S., Park P.J., Chung J.H., Kwon O.S., Kim K.H. (2012). Hair growth–promoting effects of adiponectin in vitro. J. Investig. Dermatol..

[63] Wojciechowicz K., Markiewicz E., Jahoda C.A.B. (2008). C/EBPalpha identifies differentiating preadipocytes around hair follicles in foetal and neonatal rat and mouse skin. Exp. Dermatol..

[64] Paus R., Klein J., Permana P.A., Owecki M., Chaldakov G.N., Böhm M., Hausman G., Lapière C.M., Atanassova P., Sowiński J. (2007). What are subcutaneous adipocytes really good for…?. Exp. Dermatol..

[65] Christensen S., Robinson K., Thomas S., Williams D.R. (2024). Dietary intake by patients taking GLP-1 and dual GIP/GLP-1 receptor agonists: A narrative review and discussion of research needs. Obes. Pillars..

[66] Taghizadeh N., Salhab H., Alirezaei A., Najjari K., Yang W., Abedi-Kichi R., Esparham A., Shahmiri S.S. (2025). Hair loss and metabolic and bariatric Surgery: An updated systematic review and meta-analysis. Obes. Surg..

[67] Li J., Wu H., Ma W., Yuan T., Chen X., Pan Y. (2025). Activation of cyclooxygenase-2 signaling mediates liraglutide-induced adipose lipolytic activity. Eur. J. Pharmacol..

[68] Luciani P., Fibbi B., Mazzanti B., Deledda C., Ballerini L., Aldinucci A., Benvenuti S., Saccardi R., Peri A. (2018). The effects of exendin-4 on bone marrow-derived mesenchymal cells. Endocrine.

[69] Martins F.F., Marinho T.S., Cardoso L.E.M., Barbosa-da-Silva S., Souza-Mello V., Aguila M.B., Mandarim-de-Lacerda C.A. (2022). Semaglutide (GLP-1 receptor agonist) stimulates browning on subcutaneous fat adipocytes and mitigates inflammation and endoplasmic reticulum stress in visceral fat adipocytes of obese mice. Cell Biochem. Funct..

[70] He M., Su H., Gao W., Johansson S.M., Liu Q., Wu X., Liao J., Young A.A., Bartfai T., Wang M.-W. (2010). Reversal of obesity and insulin resistance by a non-peptidic glucagon-like peptide-1 receptor agonist in diet-induced obese mice. PLoS ONE.

[71] Infante M., Ricordi C., Pacifici F., Pastore D., Infante R., Caprio M., Chiereghin F., De Stefano A., Frank G., De Rose A. (2026). Ironing out possible micronutrient deficiencies associated with incretin receptor agonist-based therapies: Proposed practical strategies to prevent and manage iron deficiency. Nutrients.

[72] Guo E.L., Katta R. (2017). Diet and hair loss: Effects of nutrient deficiency and supplement use. Dermatol. Pract. Concept..

[73] Yang J., Ren J., Song J., Liu F., Wu C., Wang X., Gong L., Li W., Xiao F., Yan F. (2013). Glucagon-like peptide 1 regulates adipogenesis in 3T3-L1 preadipocytes. Int. J. Mol. Med..

[74] Liu R., Li N., Lin Y., Wang M., Peng Y., Lewi K., Wang Q. (2016). Glucagon like peptide-1 promotes adipocyte differentiation via the Wnt4 mediated sequestering of beta-catenin. PLoS ONE.

[75] El Bekay R., Coín-Aragüez L., Fernández-García D., Oliva-Olivera W., Bernal-López R., Clemente-Postigo M., Delgado-Lista J., Diaz-Ruiz A., Guzman-Ruiz R., Vázquez-Martínez R. (2016). Effects of glucagon-like peptide-1 on the differentiation and metabolism of human adipocytes. Br. J. Pharmacol..

[76] Li Y., Du J., Zhu E., Zhang J., Han J., Zhao W., Sun B., Tian D. (2018). Liraglutide suppresses proliferation and induces adipogenic differentiation of 3T3-L1 cells via the Hippo-YAP signaling pathway. Mol. Med. Rep..

[77] Singh A., Fernandes J.R.D., Chhabra G., Krishna A., Banerjee A. (2019). Liraglutide modulates adipokine expression during adipogenesis, ameliorating obesity, and polycystic ovary syndrome in mice. Endocrine.

[78] Cantini G., Di Franco A., Samavat J., Forti G., Mannucci E., Luconi M. (2015). Effect of liraglutide on proliferation and differentiation of human adipose stem cells. Mol. Cell. Endocrinol..

[79] Liu H., Zhan Y.-L., Luo G.-J., Zou L.-L., Li Y., Lu H.-Y. (2020). Liraglutide and insulin have contrary effects on adipogenesis of human adipose-derived stem cells via Wnt pathway. Diabetes Metab. Syndr. Obes..

[80] Vaittinen M., Ilha M., Herbers E., Wagner A., Virtanen K.A., Pietiläinen K.H., Pirinen E., Pihlajamäki J. (2023). Liraglutide demonstrates a therapeutic effect on mitochondrial dysfunction in human SGBS adipocytes in vitro. Diabetes Res. Clin. Pract..

[81] Eckel R.H., Fujimoto W.Y., Brunzell J.D. (1979). Gastric inhibitory polypeptide enhanced lipoprotein lipase activity in cultured preadipocytes. Diabetes.

[82] Song D.H., Getty-Kaushik L., Tseng E., Simon J., Corkey B.E., Wolfe M.M. (2007). Glucose-dependent insulinotropic polypeptide enhances adipocyte development and glucose uptake in part through Akt activation. Gastroenterology.

[83] English A., Craig S.L., Flatt P.R., Irwin N. (2020). Individual and combined effects of GIP and xenin on differentiation, glucose uptake and lipolysis in 3T3-L1 adipocytes. Biol. Chem..

[84] Zhou B., Dong C., Zhao B., Su X., Luo Y., Xie L., Tian Y., Zhang R., Yang L. (2020). (EX-4)2-Fc, an effective long-acting GLP-1 receptor agonist, reduces obesity-related inflammation by inhibiting leptin expression. Biochem. Biophys. Res. Commun..

[85] Mostafa A.M., Hamdy N.M., El-Mesallamy H.O., Abdel-Rahman S.Z. (2015). Glucagon-like peptide 1 (GLP-1)-based therapy upregulates LXR-ABCA1/ABCG1 cascade in adipocytes. Biochem. Biophys. Res. Commun..

[86] Byun S., Sotzen M.R., Knappenberger M.A., Bento M.T., Asker M., Olekanma D.I., Skibicka K.P. (2025). Advantage of semaglutide: Comprehensive analysis of metabolic impact of semaglutide-treated and pair-fed rats. Compr. Physiol..

[87] Killion E.A., Chen M., Falsey J.R., Sivits G., Hager T., Atangan L., Helmering J., Lee J., Li H., Wu B. (2020). Chronic glucose-dependent insulinotropic polypeptide receptor (GIPR) agonism desensitizes adipocyte GIPR activity mimicking functional GIPR antagonism. Nat. Commun..

[88] Alanteet A.A., Attia H.A., Shaheen S., Alfayez M., Alshanawani B. (2021). Anti-proliferative activity of glucagon-like peptide-1 receptor agonist on obesity-associated breast cancer: The impact on modulating adipokines’ expression in adipocytes and cancer cells. Dose Response.

[89] Díaz-Soto G., de Luis D.A., Conde-Vicente R., Izaola-Jauregui O., Ramos C., Romero E. (2014). Beneficial effects of liraglutide on adipocytokines, insulin sensitivity parameters and cardiovascular risk biomarkers in patients with Type 2 diabetes: A prospective study. Diabetes Res. Clin. Pract..

[90] Nie Y., Ma R.C., Chan J.C.N., Xu H., Xu G. (2012). Glucose-dependent insulinotropic peptide impairs insulin signaling via inducing adipocyte inflammation in glucose-dependent insulinotropic peptide receptor-overexpressing adipocytes. FASEB J..

[91] Bittencourt J.O.A., Marcondes-de-Castro I.A., Marinho T.S., Aguila M.B., Mandarim-de-Lacerda C.A. (2026). Tirzepatide counteracts brown adipose tissue whitening, inflammation, and mitochondrial dysfunction in estrogen-deficient obese diabetic mice. Life Sci..

[92] Dziewońska A., Polus A., Gruca A., Solnica B., Góralska J. (2026). Irisin may be involved in exendin-4 mitochondrial action in human adipocytes. Arch. Med. Sci..

[93] Kim Chung L.T., Hosaka T., Yoshida M., Harada N., Sakaue H., Sakai T., Nakaya Y. (2009). Exendin-4, a GLP-1 receptor agonist, directly induces adiponectin expression through protein kinase A pathway and prevents inflammatory adipokine expression. Biochem. Biophys. Res. Commun..

[94] Wang A., Li T., An P., Yan W., Zheng H., Wang B., Mu Y. (2017). Exendin-4 upregulates adiponectin level in adipocytes via Sirt1/Foxo-1 signaling pathway. PLoS ONE.

[95] Pi-Sunyer X., Astrup A., Fujioka K., Greenway F., Halpern A., Krempf M., Lau D.C.W., Le Roux C.W., Ortiz R.V., Jensen C.B. (2015). A randomized, controlled trial of 3.0 mg of liraglutide in weight management. N. Engl. J. Med..

[96] Thomas M.K., Nikooienejad A., Bray R., Cui X., Wilson J., Duffin K., Milicevic Z., Haupt A., Robins D.A. (2021). Dual GIP and GLP-1 receptor agonist tirzepatide improves beta-cell function and insulin sensitivity in type 2 diabetes. J. Clin. Endocrinol. Metab..

[97] Lee C.J., Mao H., Thieu V.T., Landó L.F., Thomas M.K. (2023). Tirzepatide as monotherapy improved markers of beta-cell function and insulin sensitivity in type 2 diabetes (SURPASS-1). J. Endocr. Soc..

[98] Kim S.-J., Nian C., McIntosh C.H.S. (2007). Resistin is a key mediator of glucose-dependent insulinotropic polypeptide (GIP) stimulation of lipoprotein lipase (LPL) activity in adipocytes. J. Biol. Chem..

[99] Gutierrez A.D., Gao Z., Hamidi V., Zhu L., Saint Andre K.B., Riggs K., Ruscheinsky M., Wang H., Yu Y., Miller C. (2022). Anti-diabetic effects of GLP1 analogs are mediated by thermogenic interleukin-6 signaling in adipocytes. Cell Rep. Med..

[100] Zhang R., Yao K., Chen S., Pan X., Wu F., Gao P. (2023). Liraglutide promotes angiogenesis in adipose tissue via suppression of adipocyte-derived IL-6. Biochem. Biophys. Res. Commun..

[101] Chen S., Okahara F., Osaki N., Shimotoyodome A. (2015). Increased GIP signaling induces adipose inflammation via a HIF-1α-dependent pathway and impairs insulin sensitivity in mice. Am. J. Physiol. Endocrinol. Metab..

[102] Timper K., Grisouard J., Sauter N.S., Herzog-Radimerski T., Dembinski K., Peterli R., Frey D.M., Zulewski H., Keller U., Müller B. (2013). Glucose-dependent insulinotropic polypeptide induces cytokine expression, lipolysis, and insulin resistance in human adipocytes. Am. J. Physiol. Endocrinol. Metab..

[103] Gögebakan Ö., Osterhoff M.A., Schüler R., Pivovarova O., Kruse M., Seltmann A.-C., Mosig A.S., Rudovich N., Nauck M., Pfeiffer A.F.H. (2015). GIP increases adipose tissue expression and blood levels of MCP-1 in humans and links high energy diets to inflammation: A randomised trial. Diabetologia.

[104] Gao X., Salomon C., Freeman D.J. (2017). Extracellular vesicles from adipose tissue—A potential role in obesity and type 2 diabetes?. Front. Endocrinol..

[105] Connolly K.D., Guschina I.A., Yeung V., Clayton A., Draman M.S., Von Ruhland C., Ludgate M., James P.E., Rees D.A. (2015). Characterisation of adipocyte-derived extracellular vesicles released pre- and post-adipogenesis. J. Extracell. Vesicles.

[106] Kalinina N., Kharlampieva D., Loguinova M., Butenko I., Pobeguts O., Efimenko A., Ageeva L., Sharonov G., Ischenko D., Alekseev D. (2015). Characterization of secretomes provides evidence for adipose-derived mesenchymal stromal cells subtypes. Stem Cell Res. Ther..

[107] Simental-Mendía L.E., Sánchez-García A., Linden-Torres E., Simental-Mendía M. (2021). Effect of glucagon-like peptide-1 receptor agonists on circulating levels of leptin and resistin: A meta-analysis of randomized controlled trials. Diabetes Res. Clin. Pract..

[108] Simental-Mendía L.E., Sánchez-García A., Linden-Torres E., Simental-Mendía M. (2021). Impact of glucagon-like peptide-1 receptor agonists on adiponectin concentrations: A meta-analysis of randomized controlled trials. Br. J. Clin. Pharmacol..

[109] Kanbay M., Shah E., AlShiab R., Ozbek L., Ay S., Rustamov A., Covic A., Mallamaci F., Zoccali C. (2026). Effects of GLP-1 receptor agonists and dual GIP/GLP-1 receptor agonists on inflammatory and metabolic biomarkers in type 2 diabetes: A systematic review and meta-analysis. Diabetes Obes. Metab..

[110] Alharbi S.H. (2024). Anti-inflammatory role of glucagon-like peptide 1 receptor agonists and its clinical implications. Ther. Adv. Endocrinol. Metab..

[111] Firsowicz M., Kamrani P., Zubair R., Boen M., Dayan S.H., Fabi S.G. (2026). Cutaneous variations in stem-cell population in those on GLP1-receptor agonists: A comparative controlled study. Dermatol. Surg..

[112] Nicu C., Jackson J., Shahmalak A., Pople J., Ansell D., Paus R. (2023). Adiponectin negatively regulates pigmentation, Wnt/β-catenin and HGF/c-Met signalling within human scalp hair follicles ex vivo. Arch. Dermatol. Res..

[113] Andjelkov K., Eremin I.I., Korac A. (2022). Different levels of EGF, VEGF, IL-6, MCP-1, MCP-3, IP-10, Eotaxin, and MIP-1α in the adipose-derived stem cell secretome in androgenetic alopecia. Exp. Dermatol..

[114] Balık A.R., Balık Z.B., Aktaş A., Neşelioğlu S., Karabulut E., Karabulut A.B. (2021). Examination of androgenetic alopecia with serum biomarkers. J. Cosmet. Dermatol..

[115] Nagaev I., Bokarewa M., Tarkowski A., Smith U. (2006). Human resistin is a systemic immune-derived proinflammatory cytokine targeting both leukocytes and adipocytes. PLoS ONE.

[116] Kang D.-H., Kwon S.-H., Sim W.-Y., Lew B.-L. (2024). Telogen effluvium associated with weight loss: A single center retrospective study. Ann. Dermatol..

[117] Walker G.E., Verti B., Marzullo P., Savia G., Mencarelli M., Zurleni F., Liuzzi A., Di Blasio A.M. (2007). Deep subcutaneous adipose tissue: A distinct abdominal adipose depot. Obesity.

[118] Xu F., Lin B., Zheng X., Chen Z., Cao H., Xu H., Liang H., Weng J. (2016). GLP-1 receptor agonist promotes brown re-modelling in mouse white adipose tissue through SIRT1. Diabetologia.

[119] Góralska J., Śliwa A., Gruca A., Raźny U., Chojnacka M., Polus A., Solnica B., Malczewska-Malec M. (2017). Glucagon-like peptide-1 receptor agonist stimulates mitochondrial bioenergetics in human adipocytes. Acta. Biochim. Pol..

[120] Wang X., Chen S., Lv S., Li Z., Ren L., Zhu H., Xie X., Liu Y. (2021). Liraglutide suppresses obesity and promotes browning of white fat via miR-27b in vivo and in vitro. J. Int. Med. Res..

[121] Sun Y., Xia Y., Ge W., Li Q. (2026). Tirzepatide reduces intracellular lipid content by promoting the browning of white fat via the cAMP signaling pathway. Eur. J. Pharmacol..

[122] Model J.F.A., Normann R.S., Vogt É.L., Dentz M.V., de Amaral M., Xu R., Bachvaroff T., Spritzer P.M., Chung J.S., Vinagre A.S. (2024). Interactions between glucagon like peptide 1 (GLP-1) and estrogens regulates lipid metabolism. Biochem. Pharmacol..

[123] Chang E., Varghese M., Singer K. (2018). Gender and sex differences in adipose tissue. Curr. Diab. Rep..

[124] Chou Y.-Y., Inoue M., Toge T., Yoshimura A., Takeuchi S.Y., Takahashi O., Haraguchi K., Kawasaki K., Kaminuma O., Matsubara T. (2026). Sex differences in physiological hair cycling and adhesive material-induced hair regeneration in mice. bioRxiv.

[125] Oh H.S., Smart R.C. (1996). An estrogen receptor pathway regulates the telogen-anagen hair follicle transition and influences epidermal cell proliferation. Proc. Natl. Acad. Sci. USA.

[126] Won C.H., Yoo H.G., Kwon O.S., Sung M.Y., Kang Y.J., Chung J.H., Park B.S., Sung J.-H., Kim W.S., Kim K.H. (2010). Hair growth promoting effects of adipose tissue-derived stem cells. J. Dermatol. Sci..

[127] Zari S. (2023). Efficacy of adipocyte-derived stem cells-conditioned media in telogen effluvium. Stem Cells Cloning.

[128] Lee E., Choi M.S., Cho B.S., Won Y.J., Lee J.H., Kim S.R., Kim M.H., Jeon J.H., Park G.H., Kwon H.H. (2024). The efficacy of adipose stem cell-derived exosomes in hair regeneration based on a preclinical and clinical study. Int. J. Dermatol..

