# Supplementary material for: Adipocytes as a Mechanistic Link Between Incretin-Based Therapies and Hair Loss
Source: Biology (Basel). 2026 Sep 17;15(18):1642. doi: 10.3390/biology15181642 (PMC13604800; doi:10.3390/biology15181642)
Supplement: Supplementary file 1 [file biology-15-01642-s001.zip › biology-4543441-supplementary.pdf]

Supplementary Table S1: Clinical Studies Examining Hair Loss in Users of Incretin-Based Therapies.

Reference	Study Design	Population	Duration; Follow-Up	Drug/ Comparator	Weight Change	Absolute Event Number (Percent)	Effect Estimate	Event Rate (Years)	Covariate Adjustment
Knop, 2023 [36]	Randomized Trial	Obesity	68 Weeks; 7 Weeks	Semaglutide (N=334)	-15.5 kg; -15.1%	**Alopecia**: N=23 (7%)		56/1000	
				Placebo (N=333)	2.5 kg; 2.4%	**Alopecia**: N=9 (3%)		21/1000	
Eckard, 2024 [35]	Randomized Trial	HIV-Associated Lipohypertrophy	32 Weeks	Semaglutide (N=54)	-10.4%	**Alopecia**: N=1 (2%)			
				Placebo (N=54)		**Alopecia**: N=0 (0%)			
Danne, 2017 [41]	Randomized Trial	Obesity (12-17 years old)	5-6 Weeks; Up to 2 Weeks	Liraglutide (N=14)	-2.55 kg	**Alopecia**: N=1 (7%)			
				Placebo (N=7)	-1.85kg	**Alopecia**: N=0 (0%)			
Jastreboff, 2022 [42]	Randomized Trial	Obesity	72 Weeks; 4 Weeks	Tirzepatide, 5 mg (N=630)	-15%	**Alopecia**: N=32 (5.1%)			
				Tirzepatide, 10 mg (N=636)	-19.5%	**Alopecia**: N=31 (4.9%)			
				Tirzepatide, 15 mg (N=630)	-20.9%	**Alopecia**: N=36 (5.7%)			
				Placebo (N=643)	-3.1%	**Alopecia**: N=6 (0.9%)			
Wadden, 2023 [43]	Randomized Trial	Obesity	72 Weeks; 4 Weeks	Tirzepatide (N=287)	-18.4%	**Alopecia**: N=20 (7%)			
				Placebo (N=292)	+2.5%	**Alopecia**: N=4 (1.4%)			
Yu, 2025 [7]	Meta-Analysis	Type 2 Diabetes; Obesity		GLP-1RA (N=7318)		**Alopecia**: N=132 (1.80%)	**Alopecia** (RR): 3.40 (1.18-9.81)	6/1000	
				Placebo (N=5928)		**Alopecia**: N=16 (0.27%)		0.8/1000	
Vidal, 2026 [33]	Retrospective (TriNetX)	Unspecified		GLP-1RA (N=547,993)			**NSHL** (aOR): 1.40 (1.31-1.49); **AGA** (aOR): 1.64 (1.35-1.99); **TE** (aOR): 1.76 (1.34-2.32); No Association for AA		Demographics and Type 2 Diabetes Comorbidity
				No GLP-1RA Exposure (N=547,993)					

Reference	Study Design	Population	Duration; Follow-Up	Drug/ Comparator	Weight Change	Absolute Event Number (Percent)	Effect Estimate	Event Rate (Years)	Covariate Adjustment
Neubauer, 2025 [8]	Retrospective (TriNetX)	Obesity		GLP-1RA (N=218,147)		**TE**: N=187 (0.1%)	**TE** (HR): 1.39 (1.01-1.91) GLP1-RA vs. Other Weight Loss Drugs; No Association vs. Bariatric Surgery; **TE** (HR): 2.65 (1.32-5.32) Tirzepatide vs. Semaglutide or Liraglutide		Demographics and Comorbidities
				Other Weight Loss Drugs (N=121,383)		**TE**: N=292 (0.2%) [Combined Controls]			
				Bariatric Surgery (N=22,088)					
Ching, 2025 [6]	Retrospective (All of Us Database)	Unspecified		Semaglutide		**AGA**: N=96 (0.91%); **AA**: N= 45 (0.43%); **TE**: N=59 (0.56%); **†Other**: N=655 (6.26%)	**AGA** (OR): 1.71 (1.37-2.14); No Association for AA, TE, †Other		Demographics, Comorbidities, Haemoglobin A1C, BMI, Weight, Income, Social Determinants of Health
				Tirzepatide		**AGA**: N<20 (<0.19%); **AA**: N<20 (<0.19%); **TE**: N<20 (<0.19%); **Other**: N<20 (<0.19%)	No Association for AA, AGA, TE, Other		
				Exenatide		**AGA**: N=21 (0.20%); **AA**: N<20 (<0.19%); **TE**: N<20 (<0.19%); **Other**: N=140 (1.33%)	**AGA** (OR): 2.41 (1.52-3.82); **TE** (OR): 2.36 (1.45-3.85); **Other** (OR): 1.39 (1.15-1.67); No Association for AA		
				Liraglutide		**AGA**: N=73 (0.69%); **AA**: N=33 (0.35%); **TE**: N=38 (0.36%); **Other**: N=431 (4.12%)	**AGA** (OR): 2.26 (1.76-2.91); **Other** (OR): 1.14 (1.03-1.27); No Association for AA, TE		
				Alopecia Unrelated to GLP-1RA + GIP/GLP-1RA					
Tang, 2026 [34]	Retrospective (Penn Medicine)	Type 2 Diabetes	2.36-2.95 Years	GLP-1RA + GIP/GLP-1RA (N=11,991 and 12,030)		**Alopecia**: N=198 (1.65%) and 229 (1.90%)	**Alopecia** (HR): 1.37 (1.08-1.73) vs. SGLT-2i; 1.68 (1.28-2.20) vs. DPPi **NSA** (HR): 1.53 (1.18-1.97) vs. SGLT-2i; 1.72 (1.28-2.31) vs. DPP-4i	6.91/1000 and 6.53/1000	Demographics, Comorbidities, Socioeconomic Factors, Laboratory Values, BMI
				SGLT-2i (N=15,323)		**Alopecia**: N=182 (1.19%)		5.04/1000	

Reference	Study Design	Population	Duration; Follow-Up	Drug/ Comparator	Weight Change	Absolute Event Number (Percent)	Effect Estimate	Event Rate (Years)	Covariate Adjustment
				DPP-4i (N=11,195)		**Alopecia**: N=128 (1.14%)		3.89/1000	
Sodhi, 2025 [Pre-Print] [37]	Retrospective (IQVIA PharMetrics)	Unspecified; Diabetes Excluded		Semaglutide (N=1926)			**Hair Loss** (aHR): 2.08 (1.17-3.72) In Women; No Association in Men	26.5/1000	Demographics, Comorbidities, Steroid Use
				Bupropion-Naltrexone (N=1348)				11.8/1000	
Singal, 2025 [38]	Retrospective (TriNetX)	Type 2 Diabetes		Semaglutide (N=584,475)		**AGA**: N=797 (0.14%)	**AGA** (HR): 4.43 (3.81-5.16)		Demographics
				Liraglutide (N=212,725)		**AGA**: N=227 (0.11%)	**AGA** (HR): 2.33 (1.82-3.00)		
				Tirzepatide (N=197,335)		**AGA**: N=273 (0.14%)	**AGA** (HR): 5.67 (4.42-7.28); **AGA** (HR): 1.53 (1.29-1.82) vs. Semaglutide; **AGA** (HR): 2.50 (2.07-3.02) vs. Liraglutide		
				Metformin (N=1,448,648)		**AGA**: N=541 (0.04%)			
Jairath, 2024 [Abstract] [45]	Retrospective (Barnes Jewish Healthcare System + TriNetX)	Unspecified	6 Months	Semaglutide		**TE/NSHL**: N=0.80-0.83%			
				Placebo					
Gong, 2025 [Abstract] [46]	Retrospective (TriNetX)	Type 2 Diabetes		Semaglutide (N=13,920)			No Association		Demographics, BMI, Haemoglobin A1c
				Placebo (N=13,920)					
Godfrey, 2025 [40]	Disproportionality (FAERS)	Unspecified		Semaglutide		**Alopecia**: N=199	**Alopecia** (ROR): 2.46 (2.14-2.83)		
				Liraglutide		**Alopecia**: N=20	No Association		
				Exenatide		**Alopecia**: N=6	No Association		
				Tirzepatide		**Alopecia**: N=179	**Alopecia** (ROR): 1.73 (1.42-2.09)		
Kim, 2025 [39]	Disproportionality (VigiBase)	Unspecified		Liraglutide (N=43,917)		**Hair Loss**: N=278 (0.63%)	No Association		

Reference	Study Design	Population	Duration; Follow-Up	Drug/ Comparator	Weight Change	Absolute Event Number (Percent)	Effect Estimate	Event Rate (Years)	Covariate Adjustment
				Semaglutide (N=41,326)		**Hair Loss**: N=502 (1.21%)	**Hair Loss** (aROR): 1.42 (1.30-1.55)		
				Tirzepatide (N=31,971)		**Hair Loss**: N=271 (0.85%)	No Association		
Carmichael, 2025 [44]	Trial Exit Interviews	Obesity		Tirzepatide (N=86)		**Hair Loss/ Thinning**: N=6 (7%)			

†Other: Any Alopecia Extending Beyond AA, AGA, TE.

AA: Alopecia Areata; AGA: Androgenetic Alopecia; aHR: Adjusted Hazard Ratio; aOR: Adjusted Odds Ratio; aROR: Adjusted Reporting Odds Ratio; DPP-4i: Dipeptidyl Peptidase-4 Inhibitors; GIP: glucose-dependent insulinotropic polypeptide; GLP-1RA: Glucagon-like Peptide-1 Receptor Agonist; HR: Hazard Ratio; NSA: Non-Scarring Alopecia; NSHL: Non-Scarring Hair Loss; OR: Odds Ratio; ROR: Reporting Odds Ratio; RR: Risk Ratio; SGLT-2i: Sodium-Glucose Cotransporter 2 Inhibitors; TE: Telogen Effluvium.

Supplementary Table S2: Effects of Incretin-Based Treatments on Adipogenic Differentiation.

Reference	Model Type	Model Details	Treatment	†Key Findings
Yang, 2013 [73]	Mouse Cell Line	3T3-L1 (During differentiation)	GLP-1	• Increased PPARγ mRNA + Protein<br>• Increased C/EBPα mRNA + Protein<br>• *Increased FABP4 mRNA + Protein*<br>• *Increased LPL mRNA*
Liu, 2016 [74]	Mouse Cell Line	3T3-L1 (During differentiation)	GLP-1	• Increased Differentiation<br>• Increased PPARγ mRNA<br>• Increased C/EBPβ mRNA<br>• *Increased LPL mRNA*
El Bekay, 2016 [75]	Human Primary Cells	SVFC (During Differentiation)	GLP-1	• Decreased ADRP mRNA<br>• *Decreased FABP4 mRNA*<br>• *Increased HSL mRNA*<br>• *Increased Perilipin mRNA*
Lee, 2015 [21]	Human Primary Cells	ADSC (During Differentiation)	GLP-1	• Decreased PPARγ mRNA<br>• *Decreased LPL mRNA*<br>• *Decreased FABP4 mRNA*
Luciani, 2018 [68]	Human Primary Cells	MSC (During differentiation)	Exendin-4	• **Pre-Treatment:**<br>○ Increased Differentiation<br>○ Increased PPARγ mRNA<br>○ *Increased FABP4 mRNA*<br>• **Concurrent Treatment:**<br>○ Decreased Differentiation<br>○ Decreased PPARγ mRNA<br>○ *Decreased FABP4 mRNA*
Challa, 2012 [19]	Mouse Cell Line	3T3-L1 (Preadipocytes)	Liraglutide	• Increased Differentiation
Chen, 2017 [20]	Mouse Cell Line	3T3-L1 (Preadipocytes)	Liraglutide	• Increased Differentiation<br>• Increased PPARγ mRNA<br>• *Increased FABP4 mRNA*
Li, 2018 [76]	Mouse Cell Line	3T3-L1 (During Differentiation)	Liraglutide	• Increased Differentiation<br>• Increased PPARγ mRNA + Protein<br>• Increased C/EBPα mRNA + Protein<br>• *Increased FABP4 mRNA + Protein*
Singh, 2019 [77]	Mouse Cell Line	3T3-L1 (During differentiation)	Liraglutide	• Increased Differentiation<br>• Increased PPARγ mRNA<br>• Increased C/EBPα mRNA
Cantini, 2015 [78]	Human Primary Cells	ADSC (sWAT)	Liraglutide	• Decreased Differentiation<br>• *Decreased FABP4 mRNA*
Liu, 2020 [79]	Human Primary Cells	ADSC (sWAT)	Liraglutide	• Decreased Differentiation<br>• Decreased PPARγ mRNA<br>• Decreased C/EBPα mRNA
Vaittinen, 2023 [80]	Human Cell Line	SGBS (During differentiation)	Liraglutide	• *Increased HSL mRNA*<br>• *Increased PNPLA2 mRNA*
Eckel, 1979 [81]	Mouse Cell Line	3T3-L1 (Preadipocytes)	GIP	• *Increased LPL Protein Secretion*

Reference	Model Type	Model Details	Treatment	†Key Findings
Song, 2007 [82]	Mouse Cell Line	3T3-L1 (During differentiation)	GIP	• *Increased FABP4 mRNA*
English, 2020 [83]	Mouse Cell Line	3T3-L1 (During differentiation)	(D-ALA^2)-GIP	• Increased Differentiation<br>• *Increased ATGL mRNA*<br>• *Increased LPL mRNA*<br>• *Decreased HSL mRNA*

†Mature adipocyte genes are italicized.

ADRP: adipocyte differentiation-related protein; ADSC: adipose-derived stem cell; ATGL: adipose triglyceride lipase; C/EBP: CCAAT/enhancer-binding protein; FABP4: fatty acid-binding protein 4; GIP: glucose-dependent insulinotropic polypeptide; GLP-1: glucagon-like peptide-1; HSL: hormone-sensitive lipase; LPL: lipoprotein lipase; MSC: mesenchymal stromal cell; PNPLA2: patatin-like phospholipase domain containing-2; PPARγ: peroxisome proliferator-activation receptor gamma; SGBS: Simpson Golabi Behmen Syndrome; SVFC: stromal vascular fraction cells; sWAT: subcutaneous white adipose tissue.

Supplementary Table S3: Effect of Incretin-Based Treatments on Leptin.

Reference	Model Type	Model Details	Treatment	Key Findings
He, 2010 [70]	Mouse Model (Obesity)	Systemic/ Circulating	Boc5	• Decreased Leptin Protein
Zhou, 2020 [84]	Mouse Model (Obesity)	Systemic/ Circulating	(Ex-4)$_2$-Fc	• Decreased Leptin Protein
	Mouse Cell Line	3T3-L1 (Differentiated)	(Ex-4)$_2$-Fc	• Decreased Leptin mRNA
Mostafa, 2015 [85]	Mouse Cell Line	3T3-L1 (Differentiated)	Exendin-4	• Decreased Leptin mRNA + Protein
Byun, 2025 [86]	Rat Model (Obesity)	Systemic/ Circulating	Semaglutide	• Decreased Leptin Protein
Martins, 2022 [69]	Mouse Model (Obesity)	Systemic/ Circulating	Semaglutide	• Decreased Leptin Protein
Killion, 2020 [87]	Mouse Model (Obesity)	Systemic/ Circulating	Liraglutide	• Decreased Leptin Protein
Alanteet, 2021 [88]	Human sWAT (Obesity)	ADSCs	Liraglutide	• Decreased Leptin mRNA + Protein
Díaz-Soto, 2014 [89]	Human (Obesity + Diabetes)	Systemic/ Circulating	Liraglutide	• No Difference in Leptin Protein
Nie, 2012 [90]	Mouse Cell Line	3T3-L1 CAR (Differentiated)	GIP	• Decreased Leptin mRNA
Bittencourt, 2026 [91]	Mouse Model (Obesity + Diabetes)	Systemic/ Circulating	Tirzepatide	• Decreased Leptin Protein

ADSCs: adipose-derived stem cells; GIP: glucose-dependent insulinotropic polypeptide; sWAT: subcutaneous white adipose tissue.

Supplementary Table S4: Effect of Incretin-Based Treatments on Adiponectin.

Reference	Model Type	Model Details	Treatment	Key Findings
El Bekay, 2016 [75]	Human Primary Cells	SVFC-Adipocytes (Differentiated)	GLP-1	• Decreased Adiponectin mRNA
	Human sWAT	Adipocytes (Differentiated)	GLP-1	• Increased Adiponectin mRNA
	Human sWAT (Obesity)	sWAT Explants	GLP-1	• No Difference in Adiponectin mRNA
Dziewońska, 2026 [92]	Human Cell Line	CHUB-S7 (Differentiated)	Exendin-4	• No Difference in Adiponectin Secretion
Kim Chung, 2009 [93]	Mouse Cell Line	3T3-L1 (Differentiated)	Exendin-4	• Increased Adiponectin mRNA + Secretion
Wang, 2017 [94]	Mouse Cell Line	3T3-L1 (Differentiated)	Exendin-4	• Increased Adiponectin mRNA + Protein
	Mouse Model (Diabetes)	Adipose Tissue	Exendin-4	• Increased Adiponectin mRNA + Protein
He, 2010 [70]	Mouse Model (Obesity)	Systemic/ Circulating	Boc5	• Increased Adiponectin Protein
Martins, 2022 [69]	Mouse Model (Obesity)	Systemic/ Circulating	Semaglutide	• Increased Adiponectin Protein
Byun, 2025 [86]	Rat Model (Obesity)	Systemic/ Circulating	Semaglutide	• Decreased Adiponectin Protein (Males)
Cantini, 2015 [78]	Human sWAT	ADSC	Liraglutide	• Increased Adiponectin mRNA
Singh, 2019 [77]	Mouse Cell Line	3T3-L1 (Differentiated)	Liraglutide	• Increased Adiponectin Protein
Pi-Sunyer, 2015 [95]	Human (Obesity)	Systemic/ Circulating	Liraglutide	• Increased Adiponectin Protein
Díaz-Soto, 2014 [89]	Human (Diabetes, Obesity)	Systemic/ Circulating	Liraglutide	• No Difference in Adiponectin Protein
Alanteet, 2021 [88]	Human sWAT (Obesity)	ADSC	Liraglutide	• Increased Adiponectin mRNA and Protein
Killion, 2020 [87]	Mouse Model (Obesity)	Systemic/ Circulating	Liraglutide	• No Difference in Adiponectin Protein
Li, 2025 [67]	Mouse Cell Line	3T3-L1 (Differentiated)	Liraglutide	• Decreased Adiponectin Protein
Nie, 2012 [90]	Mouse Cell Line	3T3-L1 CAR (Differentiated)	GIP	• Decreased Adiponectin mRNA
Bittencourt, 2026 [91]	Mouse Model (Obesity, Diabetes)	Systemic/ Circulating	Tirzepatide	• Increased Adiponectin Protein

Reference	Model Type	Model Details	Treatment	Key Findings
Thomas, 2021 [96]	Human (Diabetes)	Systemic/ Circulating	Tirzepatide	• Increased Adiponectin Protein
Lee, 2023 [97]	Human (Diabetes)	Systemic/ Circulating	Tirzepatide	• Increased Adiponectin Protein

ADSC: adipose-derived stem cells; GIP: glucose-dependent insulinotropic polypeptide; GLP-1: glucagon-like peptide-1; SVFC: Stromal Vascular Fraction Cells; sWAT: Subcutaneous White Adipose Tissue.

Supplementary Table S5: Effects of Incretin-Based Treatments on Resistin and Visfatin.

Reference	Model Type	Model Details	Treatment	Key Findings
Martins, 2022 [69]	Mouse Model (Obesity)	Systemic/ Circulating	Semaglutide	• Decreased Resistin Protein
Byun, 2025 [86]	Rat Model (Obesity)	Systemic/ Circulating	Semaglutide	• Increased Resistin Protein (Females)
Killion, 2020 [87]	Mouse Model (Obesity)	Systemic/ Circulating	Liraglutide	• Decreased Resistin Protein
Díaz-Soto, 2014 [89]	Human (Diabetes, Obesity)	Systemic/ Circulating	Liraglutide	• Increased Resistin Protein
Kim, 2007 [98]	Mouse Cell Line	3T3-L1 (Differentiated)	GIP	• Increased Resistin Protein Secretion
Bittencourt, 2026 [91]	Mouse Model (Obesity + Diabetes)	Systemic/ Circulating	Tirzepatide	• Decreased Resistin Protein
Liu, 2013 [22]	Mouse Cell Line	3T3-L1 (Differentiated)	GLP-1	• Increased Visfatin mRNA + Protein Secretion
Dziewońska, 2026 [92]	Human Cell Line	CHUB-S7 (Differentiated)	Exendin-4	• Increased Visfatin mRNA + Protein Secretion
Díaz-Soto, 2014 [89]	Human (Diabetes, Obesity)	Systemic/ Circulating	Liraglutide	• Increased Visfatin Protein

GIP: glucose-dependent insulinotropic polypeptide; GLP-1: glucagon-like peptide-1.

Supplementary Table S6: Effects of Incretin-Based Treatments on Inflammatory Cytokines.

Reference	Model Type	Model Details	Treatment	Key Findings
Mostafa, 2015 [85]	Mouse Cell Line	3T3-L1 (Mature Adipocytes)	Exendin-4	• Decreased IL-6 mRNA + Protein
Kim Chung, 2009 [93]	Mouse Cell Line	3T3-L1 (Differentiated)	Exendin-4	• Decreased IL-6 mRNA
Gutierrez, 2022 [99]	Human (Pre- Diabetes)	Systemic/ Circulating	Exenatide	• Increased IL-6 Protein
El Bekay, 2016 [75]	Human Tissue (Diabetes + Obesity)	sWAT Biopsy	Exenatide	• Decreased IL-6 mRNA
Byun, 2025 [86]	Rat Model (Obesity)	sWAT	Semaglutide	• Increased IL-6 mRNA (Females)
		Systemic/ Circulating	Semaglutide	• No Difference in IL-6 Protein
Singh, 2019 [77]	Mouse Cell Line	3T3-L1 (Differentiated)	Liraglutide	• Increased IL-6 Protein
Zhang, 2023 [100]	Mouse Primary Cells	SVFC-Adipocytes (Differentiated)	Liraglutide	• Decreased IL-6 mRNA
Gutierrez, 2022 [99]	Mouse Model (Obesity)	Systemic/ Circulating	Liraglutide	• Increased IL-6 Protein
Alanteet, 2021 [88]	Human sWAT (Obesity)	ADSC	Liraglutide	• Decreased IL-6 Protein
Díaz-Soto, 2014 [89]	Human (Diabetes, Obesity)	Systemic/ Circulating	Liraglutide	• No Difference in IL-6 Protein
Chen, 2015 [101]	Mouse Cell Line	3T3-L1 (Differentiated)	GIP	• Increased IL-6 mRNA + Protein
Nie, 2012 [90]	Mouse Cell Line	3T3-L1 CAR (Differentiated)	GIP	• Increased IL-6 mRNA + Protein
Timper, 2013 [102]	Human sWAT (Healthy; Obesity)	SVFC-Adipocytes (Differentiated)	GIP	• Increased IL-6 mRNA
Gögebakan, 2015 [103]	Human Primary Cells (Obesity)	sWAT Biopsy	GIP Infusion	• Increased IL-6 mRNA
Martins, 2022 [69]	Mouse Model (Obesity)	Systemic/ Circulating	Semaglutide	• Decreased TNFα Protein
Byun, 2025 [86]	Rat Model (Obesity)	sWAT	Semaglutide	• Increased TNFα mRNA (Females; vs. Control)<br>• Decreased TNFα mRNA (Females; vs. Pair-Fed)
		Systemic/ Circulating	Semaglutide	• No Difference in TNFα Protein
El Bekay, 2016 [75]	Human Tissue (Diabetes + Obesity)	sWAT Biopsy	Liraglutide	• Decreased TNFα mRNA

Reference	Model Type	Model Details	Treatment	Key Findings
Alanteet, 2021 [88]	Human sWAT (Obesity)	ADSC	Liraglutide	• Decreased TNFα Protein
Díaz-Soto, 2014 [89]	Human (Diabetes, Obesity)	Systemic/ Circulating	Liraglutide	• No Difference in TNFα Protein
Zhang, 2023 [100]	Mouse Primary Cells	SVFC-Adipocytes (Differentiated)	Liraglutide	• No Difference in TNFα mRNA
Nie, 2012 [90]	Mouse Cell Line	3T3-L1 CAR (Differentiated)	GIP	• Increased TNFα mRNA
Timper, 2013 [102]	Human sWAT (Healthy; Obesity)	SVFC-Adipocytes (Differentiated)	GIP	• No Difference in TNFα mRNA
Bittencourt, 2026 [91]	Mouse Model (Diabetes + Obesity)	Systemic/ Circulating	Tirzepatide	• Decreased TNFα Protein
Kim Chung, 2009 [93]	Mouse Cell Line	3T3-L1 (Differentiated)	Exendin-4	• Decreased MCP-1 mRNA
Byun, 2025 [86]	Rat Model (Obesity)	sWAT	Semaglutide	• No Difference in MCP-1 mRNA
Martins, 2022 [69]	Mouse Model (Obesity)	Systemic/ Circulating	Semaglutide	• Decreased MCP-1 Protein
Zhang, 2023 [100]	Mouse Primary Cells	SVFC-Adipocytes (Differentiated)	Liraglutide	• No Difference in MCP-1 mRNA
Chen, 2015 [101]	Mouse Cell Line	3T3-L1 (Differentiated)	GIP	• Increased MCP-1 mRNA + Protein
Nie, 2012 [90]	Mouse Cell Line	3T3-L1 CAR (Differentiated)	GIP	• Increased MCP-1 mRNA + Protein
Timper, 2013 [102]	Human sWAT (Healthy; Obesity)	SVFC-Adipocytes (Differentiated)	GIP	• No Difference in MCP-1 mRNA
Gögebakan, 2015 [103]	Human Primary Cells (Obesity)	sWAT Biopsy	GIP Infusion	• Increased MCP-1 mRNA + Protein
Bittencourt, 2026 [91]	Mouse Model (Diabetes + Obesity)	Systemic/ Circulating	Tirzepatide	• Decreased MCP-1 Protein

ADSC: adipose-derived stem cells; GIP: glucose-dependent insulinotropic polypeptide; IL-6: interleukin-6; MCP-1: monocyte chemoattractant protein-1; SVFC: stromal vascular fraction cells; sWAT: subcutaneous white adipose tissue; TNFα: tumor necrosis factor-alpha.
